# Dissecting the role of H3K27 acetylation and methylation in PRC2 mediated control of cellular identity

**DOI:** 10.1038/s41467-019-09624-w

**Published:** 2019-04-11

**Authors:** Elisa Lavarone, Caterina M. Barbieri, Diego Pasini

**Affiliations:** 1 IEO European Institute of Oncology IRCCS, Department of Experimental Oncology, Via Adamello 16, 20139 Milan, Italy; 2 University of Milan, Department of Health Sciences, Via A. di Rudinì, 8, 20142 Milan, Italy

## Abstract

The Polycomb repressive complexes PRC1 and PRC2 act non-redundantly at target genes to maintain transcriptional programs and ensure cellular identity. PRC2 methylates lysine 27 on histone H3 (H3K27me), while PRC1 mono-ubiquitinates histone H2A at lysine 119 (H2Aub1). Here we present engineered mouse embryonic stem cells (ESCs) targeting the PRC2 subunits EZH1 and EZH2 to discriminate between contributions of distinct H3K27 methylation states and the presence of PRC2/1 at chromatin. We generate catalytically inactive EZH2 mutant ESCs, demonstrating that H3K27 methylation, but not recruitment to the chromatin, is essential for proper ESC differentiation. We further show that EZH1 activity is sufficient to maintain repression of Polycomb targets by depositing H3K27me2/3 and preserving PRC1 recruitment. This occurs in the presence of altered H3K27me1 deposition at actively transcribed genes and by a diffused hyperacetylation of chromatin that compromises ESC developmental potential. Overall, this work provides insights for the contribution of diffuse chromatin invasion by acetyltransferases in PRC2-dependent loss of developmental control.

## Abstract (web summary)

Polycomb repressive complexes PRC1 and PRC2 act non-redundantly at target genes to regulate transcription. Here the authors present engineered mouse ESCs targeting the PRC2 subunits EZH1 and EZH2 to discriminate between contributions of distinct H3K27 methylation states and the presence of PRC2/1 at chromatin, and provide evidence for the role of H3K27 acetylation in PRC2-mediated functions.

## Introduction

The proper establishment and regulation of transcriptional programs is of fundamental importance during development. Polycomb group (PcG) proteins act as epigenetic regulators that ensure the maintenance of cell-specific transcriptional programs by exerting a crucial role during establishment of cellular identity and cell fate transitions. This is guaranteed by the activity of two major PcG repressive complexes (PRCs), PRC1 and PRC2, that act non-redundantly at the same target genes to ensure proper gene repression via post-translational modifications of histone proteins^1^. The importance of PRCs is highlighted by the early embryonic lethality of knockout mice^2–5^, as well as the failure to establish proper in vitro differentiation of embryonic stem cells (ESCs) lacking core PRC1 and PRC2 subunits^6–9^. PRCs maintain a critical role also in adult life, with both PRC1 and PRC2 activities playing specific roles in controlling cell identity and tissue homeostasis^1^. Importantly, these same activities are also frequently deregulated in different type of human tumors by genetic lesions that preferentially target PRC2 activity^1^. Such mutations can result in either gain- or loss-of-function of PRC2, depending on the tissue and the environmental context^8,10–15^.

PRC1 is responsible for the deposition of histone H2A lysine 119 mono-ubiquitination (H2Aub1), catalyzed by activity of its redundant E3-ligase subunits RING1A or RING1B^16^. PRC2 activity relies on the methyltransferases EZH1 and EZH2, which deposit all three methylation states of lysine 27 onto histone H3 (H3K27me)^17–19^. EZH1 and EZH2 are mutually exclusive within PRC2 and retain distinct enzymatic proprieties in vitro, with EZH2 showing higher methyltransferase efficiency under the same reaction conditions^19^. While loss of EZH1 is dispensable for embryogenesis and mice viability^20^, EZH1 fails to compensate EZH2 loss of function that results in early embryonic lethality during gastrulation^2^. Both EZH1 and EZH2 are expressed in mouse ESCs and are found associated within PRC2^3,18^. PRC2-EZH1 and PRC2-EZH2 complexes are associated to a set of ancillary proteins that are not required for intrinsic PRC2 enzymatic activity but play distinct roles in regulating chromatin recruitment and activity^21–26^.

The mechanisms by which PRC1 and PRC2 complexes are recruited to, and stabilized at, chromatin still remain points of discussion. In *Drosophila*, recruitment occurs via Polycomb response elements (PRE), which are distal cis-regulatory elements about a few hundred base pairs long that are devoid of nucleosomes^27^. The recruitment mechanisms in mammals appear to be divergent and are still not clear. CpG islands attract PRC1 and PRC2 complexes, and several subunits, such JARID2 and Polycomb-like proteins (e.g., PHF1, MTF2, and PHF19), have direct binding affinities to these DNA motifs^28,29^; how specificity is acquired, however, remains an open issue. PRC2 has been proposed to be actively excluded from CpG islands (CpGi) by RNA polymerase II activity, which would imply that PRC2 has the potential ability to bind CpGs by default^30^. In this scenario, neither the role that H2Aub1 exerts in vivo on PRC2 recruitment, nor the role that PRC2 exerts on PRC1 recruitment, is clearly defined.

Multiple PRC1 subcomplexes with distinct biochemical proprieties have been demonstrated to exist. These complexes are commonly referred to as canonical or non-canonical, based on their dependence on H3K27me3 for recruitment to target loci^31^. It was also shown that PRC2 retains the ability to bind H2Aub1, suggesting that deposition of H2Aub1 can control PRC2 recruitment and/or stabilization at target loci^32–34^. Studies in *Drosophila melanogaster* have elegantly shown that preventing H2Aub1 deposition by mutating K117, K118, K121, and K122 of histone H2A, or by expressing a dRING catalytically inactive mutant, did not result in homeotic transformations^35^. Similarly, inactivating point mutations of RING1B in mice postpones embryonic lethality from embryonic day E10.5 to E15.5^36^. Although RING1A was still expressed in these mice, these results strongly suggest that lack of H2Aub1 deposition cannot phenocopy loss of PRC2 activity in vivo. Importantly, substituting lysine for arginine in H3 (H3K27R) resulted in homeotic transformations identical to E(z) loss-of-function in the developing fly embryos, pointing to H3K27me3 as the central hub for PcG functions in fly development^37^. Whether this also applies to mammalian development remains to be addressed.

PRC2 activity controls all forms of H3K27 methylation^17^. We have previously reported that while mono-methylated H3 (H3K27me1) is preferentially deposited at highly transcribed gene bodies, tri-methylated H3 (H3K27me3) is deposited at promoter regions concomitantly with PRC2 and PRC1 association^17^. Like the CBX proteins of canonical PRC1, EED can bind H3K27me3 with its WD40 domain to stabilize PRC2 at its target sites and to allosterically stimulate PRC2 enzymatic activity^38^. This also generates an intrinsic biochemical competition for this modification between PRC1 and PRC2; binding of PRC2 reinforces its activity at target sites but also serves as a docking site for canonical PRC1.

In contrast, di-methylated H3 (H3K27me2) is a broadly diffused modification that, in ESCs, covers 70% of total H3^17^. Essentially, H3K27me2 “fills the gaps” between H3K27me1 and H3K27me3 chromatin domains in intergenic and non-transcribed intragenic genomic space. Importantly, we and others have previously reported that global loss of H3K27 methylations results in an aberrant accumulation of H3K27 acetylation mediated by CBP and p300 activity^39,40^. This occurs at all sites in which H3K27 methylation is lost, including hyperacetylation of PcG bound promoters and non-lineage-specific enhancer elements^17^. This observation raises the question of whether early developmental failure in the absence of PRC2 activity is primarily a consequence of loss of control of promoter repression due to lack of H3K27me3 deposition, or a general failure caused by diffuse chromatin hyperacetylation due to a lack of H3K27me2 deposition.

To address these questions, we generate a set of mutants to entirely delete EZH1 and EZH2 proteins or completely inactivate EZH2 catalytic activity without altering PRC2 assembly. We now demonstrate that PRC2 is recruited to target sites independently of H3K27me3 and H2Aub1 deposition. We further show that the activity of EZH1 is sufficient to deposit H3K27me3 at all PRC2 target sites but is unable to spread this modification to neighboring chromatin. Although this is sufficient to fully recruit PRC1 activity at these sites and to prevent hyperacetylation of all PcG target promoters, EZH1 activity is unable to counteract diffused chromatin H3K27ac hyperacetylation caused by H3K27me2 loss or to support proper ESC differentiation. Together, these data strongly suggest that early developmental failure induced by loss of PRC2 activity is a consequence of chromatin H3K27 hyperacetylation rather than specific loss of repressive control at target genes.

## Results

### Generation of EZH1/2 full KO and catalytically inactive ESC

**Fig. 1:**
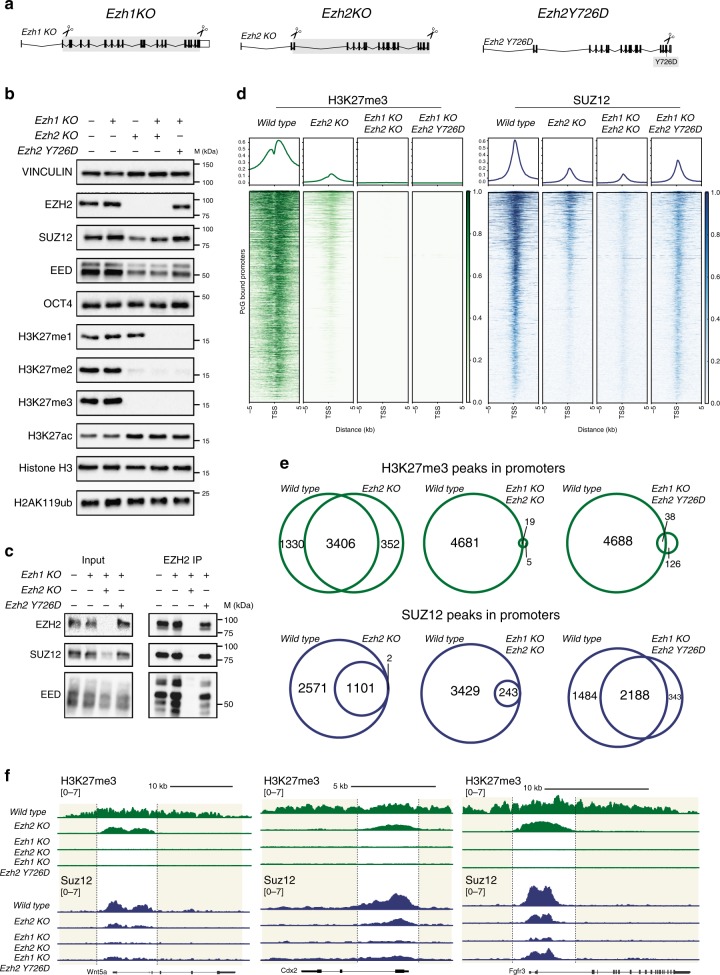
PRC2 enzymatic activity is not required for its association to chromatin. **a** Schematic representation of the CRISPR/Cas9 strategies used to generate knock-out (KO) and knock-in (KI) mESCs. Scissors indicate the position of the sgRNAs used for Cas9 targeting. **b** Western blot analysis using the indicated antibodies with protein extracts obtained from WT, *Ezh1* KO, *Ezh2* KO, *Ezh1/2* dKO, and *Ezh1* KO-*Ezh2* Y726D mouse ESC lines. Vinculin and histone H3 were used as loading controls. **c** Immunoprecipitations using EZH2 antibody with total protein extracts from the indicated cell lines. Western blots were performed with the indicated antibodies. **d** Heatmaps representing spike-in normalized H3K27me3 and normalized SUZ12 ChIP-seq intensities ± 5 kb around the transcription start sites (TSS) of the Polycomb-bound promoters in the indicated cell lines. Promoters were ranked according to their ChIP-seq intensities in WT mESCs. Enrichment plots representing the average distribution of H3K27me3 and SUZ12 ± 5 kb around TSS are shown in the upper panels. Target regions (*N* = 3968) were selected considering SUZ12 and RING1B peaks with *P* ≥ 1 × 10^–1^ in wild-type mESCs. **e** Overlap between the H3K27me3- and SUZ12-bound promoters in the indicated cell lines. **f** Representative genomic snapshots for H3K27me3 and SUZ12 ChIP-seq analyses performed in the indicated cell lines. Regions of H3K27me3 spreading outside the boundaries of SUZ12 chromatin association are highlighted

In order to dissect the role of H3K27 methylation from the assembly and recruitment of the PRC2 complex to chromatin, we generated a set of isogenic ESC lines carrying different mutations by using CRISPR/Cas9 engineering to target the PRC2 complex. Until now, EZH2-null ESC lines have used C-terminal deletions to create functionally dead EZH2 protein by eliminating its catalytic SET domain. However, this results in the expression of a truncated protein that can still assemble into a normal PRC2 complex^18,41^. To bypass this problem, we generated macrodeletions that eliminate the production of any polypeptide from the *Ezh2* gene (*Ezh2* knockout [KO]). Following a similar strategy, we have also generated a macro-deletion that targets independently the *Ezh2* paralog *Ezh1* (*Ezh1* KO). To further create double-KO (dKO) ESCs, we sequentially targeted *Ezh1* and *Ezh2* with the same gRNAs (*Ezh1/2* dKO; Fig. 1a, Supplementary Fig. 1A, B, D, and Supplementary Table 1). Consistent with previous reports, ESCs lacking PRC2 activity proliferated as wild-type (WT), with no changes in the expression of the pluripotency marker POU5F1 (OCT4; Fig. 1b). While loss of EZH1 was fully compensated by EZH2 activity, *Ezh2* KO displayed a global loss of both H3K27me2 and H3K27me3 but retained normal H3K27me1 deposition. Consistent with this, all three H3K27 methylation forms were lost in *Ezh1/2* dKOs (Fig. 1b). Overall, these data show that: (i) all three forms of H3K27 methylation are under EZH1 and EZH2 control in ESCs; (ii) H3K27me1 is fully compensated by PRC2-EZH1; and (iii) loss of H3K27me2/me3 always correlates with increased H3K27ac levels.

To further distinguish the role of the catalytic activity of PRC2 from that of its physical association at chromatin in living cells, we screened several EZH2 mutations to determine how they affected both roles. We found that the tyrosine-to-aspartic acid substitution at amino acid 731 (Y731D) completely impaired EZH2 catalytic activity. We thus generated physiological *Ezh2* Y726D (equivalent to Y731D in human) homozygous mutations in *Ezh1* KO ESCs using CRISPR/Cas9 (termed *Ezh2* Y726D; Supplementary Fig. 1C, D). This led to complete loss of all three H3K27 methylation states, to the same levels observed for *Ezh1/2* dKO (Fig. 1b). It is important to highlight that, in contrast to *Ezh1/2* dKO ESCs, SUZ12 and EED were not destabilized in *Ezh2* Y726D ESCs (Fig. 1b), and that they bound to the EZH2 Y726D mutant with an identical efficiency as in WT ESCs (Fig. 1c). Moreover, different PRC2 ancillary subunits: JARID2, MTF2, PHF19 and EPOP, which specify distinct forms of PRC2, also followed a similar expression behavior (Supplementary Fig. 2A). Overall, these data show that EZH2 Y726D mutation leads to PRC2 enzymatic loss-of-function without affecting protein stability or PRC2 physiological complex assembly.

### PRC2-EZH1 fails to spread H3K27me3 at PRC2 targets

To further explore the effects of specific EZH1 and EZH2 loss-of-function and PRC2 inactivation at a locus-specific level, we performed H3K27me3 ChIP-seq analysis, with spiked Drosophila chromatin reference for signal normalization, in the different mutant ESC lines. *Ezh1/2* dKO and *Ezh2* Y726D ESCs displayed a complete genome-wide loss of H3K27me3 deposition at PcG bound sites defined by association of PRC2 (SUZ12) or PRC1 (RING1B) in wild-type ESC (Supplementary Fig. 2B), further underscoring that the EZH2 Y726D mutation is catalytically dead (Fig. 1d). Importantly, while SUZ12 binding was displaced from chromatin in *Ezh1/2* dKO ESCs, its binding was retained in *Ezh2* Y726D ESCs, demonstrating that specific PRC2 association to target genes is independent from H3K27me3 deposition (Fig. 1d). Although bulk levels of H3K27me3 seemed to be abolished in *Ezh2* KO ESCs analyzed by western blot (Fig. 1b), ChIP-seq analysis revealed significant H3K27me3 deposition at PRC2 bound sites as compared to *Ezh1/2* dKOs or *Ezh2* Y726D ESCs (Fig. 1d, e). While SUZ12 levels were destabilized in the absence of EZH2 (Fig. 1b), residual H3K27me3 deposition also correlated with proper SUZ12 association at PRC2 sites in *Ezh2* KO ESCs (Fig. 1d, e and Supplementary Fig. 2C) displaying specific promoter localization (Fig. 1d, e and Supplementary Fig. 2C, D). Indeed, while nearly no H3K27me3 peaks were detected in *Ezh1/2* dKO*s* or *Ezh2* Y726D ESCs, specific loss of EZH2 left intact >70% of the H3K27me3-decorated sites detected in WT ESCs. This is consistent with EZH1 being specifically recruited at the same genomic sites in absence of EZH2 when re-expressed in *Ezh1/2* dKOs (Supplementary Fig. 3A–C). Importantly, MTF2, JARID2, and PHF19 were also normally recruited at target promoters in the absence of EZH2 (Supplementary Fig. 4A–C), demonstrating that EZH1 can recruit both PRC2 forms to target loci. Only the recruitment of EPOP was affected in the absence of EZH2 (Supplementary Fig. 4D), suggesting a more specific interaction with the PRC2-EZH2 complex. Genomic snapshots of representative target loci confirmed this result and further suggested that, in the absence of EZH2, H3K27me3 deposition was confined within the borders of SUZ12 peaks, with no signs of 5′ or 3′ spreading present in WT cells (Fig. 1f). Although this could be a consequence of a reduced occupancy and activity (Fig. 1b, d), this result might also suggest that the PRC2-EZH1 complex is unable to spread the H3K27me3 mark outside the area of physical PRC2 interaction with chromatin.

**Fig. 2:**
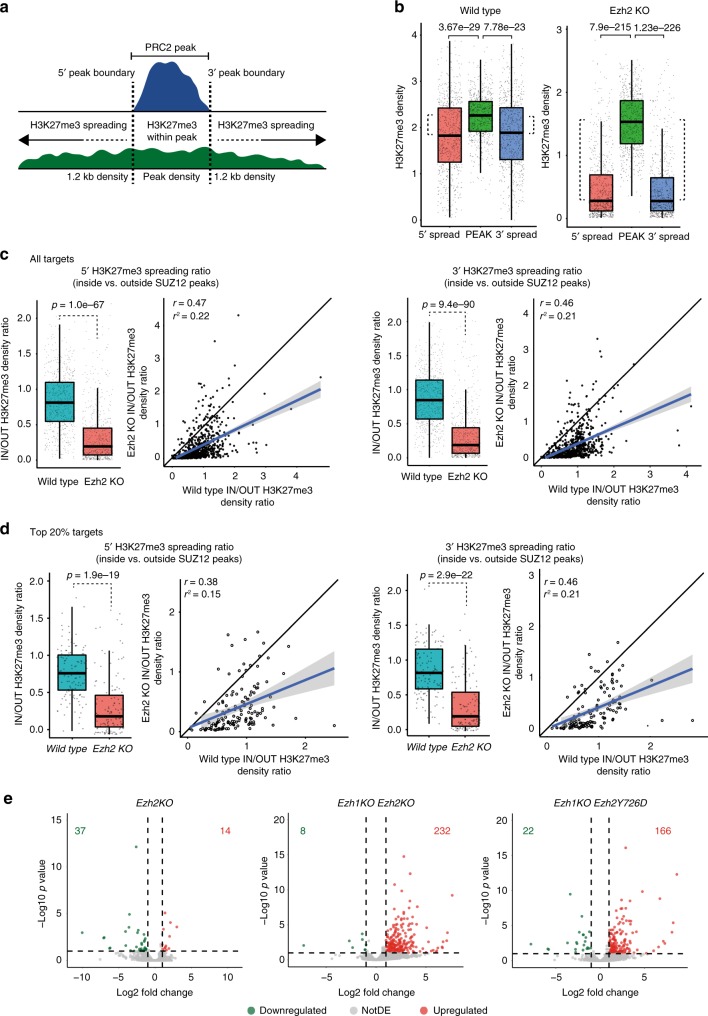
PRC2-EZH1 is unable to spread H3K27me3 deposition but preserves target genes repression. **a** Illustration describing the criteria used to select SUZ12 inside peak regions, its boundaries, and the regions selected for the analysis at the 5′ and 3′ outside SUZ12 peak boundaries. **b** Boxplots representing H3K27me3 density distribution in WT and *Ezh2* KO cells within PRC2 peaks, as well as 1.2 kb outside the 5′ and 3′ ends. *P*-values were determined using a Student’s *t*-test. **c** Left panels, boxplots representing the distribution of the H3K27me3 density ratio between the H3K27me3 density inside PRC2 peaks (PEAK in **a**) and at 5′ (left panel) or 3′ (right panels) spreading regions (5′ or 3′ spread in **a**). *P*-values were determined using a Student’s *t*-test. Right panels, the correlation for the IN/OUT H3K27me3 density ratio between WT and *Ezh2* KO cells. The linear correlation coefficient (*r*) and coefficient of determination (*r*^2^) are shown in the graphs. **d** Left panels, boxplots representing the distribution of the H3K27me3 density ratio between the H3K27me3 density inside PRC2 peaks (PEAK in **a**) and at 5′ (left panel) or 3′ (right panels) spreading regions (5′ or 3′ spread in **a**) considering the top 20% H3K27me3 enriched promoters in *Ezh2* KO cells based on *P*-values. *P*-values were determined using a Student’s *t*-test. Right panels, the correlation for the IN/OUT H3K27me3 density ratio between WT and *Ezh2* KO cells. The linear correlation coefficient (*r*) and coefficient of determination (*r*^2^) are shown in the graphs. **e** Volcano plots of differentially expressed genes in the indicated cell lines compared to WT. Log2FC ≥ 1

To gain further insight into this mechanism, we computed the density of H3K27me3 deposition inside each SUZ12 peak or within the neighboring regions (Supplementary Fig. 5A) selecting a 1.2 kb region at both ends (Fig. 2a). This analysis showed that H3K27me3 deposition is subjected to a larger intensity drop in *Ezh2* KO ESCs than in WT ESCs, suggesting a lack of H3K27me3 spreading in *Ezh2* KO ESCs (Fig. 2b). We therefore quantified the amount of spreading in WT and *Ezh2* KO ESC as the ratio between the H3K27me3 density outside compared to inside each SUZ12 peak at either the 5′ or 3′ end. Consistent with the previous result, the spreading ratio was close to 1 at both ends in WT ESCs but was significantly reduced in *Ezh2* KO ESCs, demonstrating the failure to deposit H3K27me3 outside SUZ12 peak boundaries in these cells (Fig. 2c). Similar results were obtained when the top 20% of bound sites or a larger 4 kb window is taken into consideration in the analysis (Fig. 2d and Supplementary Fig. 5B–D). Overall, these data show that PRC2-EZH1 is recruited to PcG target loci, where it deposits H3K27me3 as normal but is impaired in consolidating H3K27me3 spreading around the site of the PRC2 contact with chromatin. Importantly, although H3K27me3 spreading was compromised in absence of EZH2, EZH1 activity was sufficient to preserve target genes repression (Fig. 2e). While *Ezh1/2* dKO*s* and *Ezh2* Y726D ESC showed preferential de-repression of a common set of direct targets (Fig. 2e), these genes were maintained repressed in *Ezh2* KO with very few expression changes (Fig. 2e and Supplementary Fig. 5E).

### De novo PRC2 recruitment is independent of H3K27me3 and H2Aub1

**Fig. 3:**
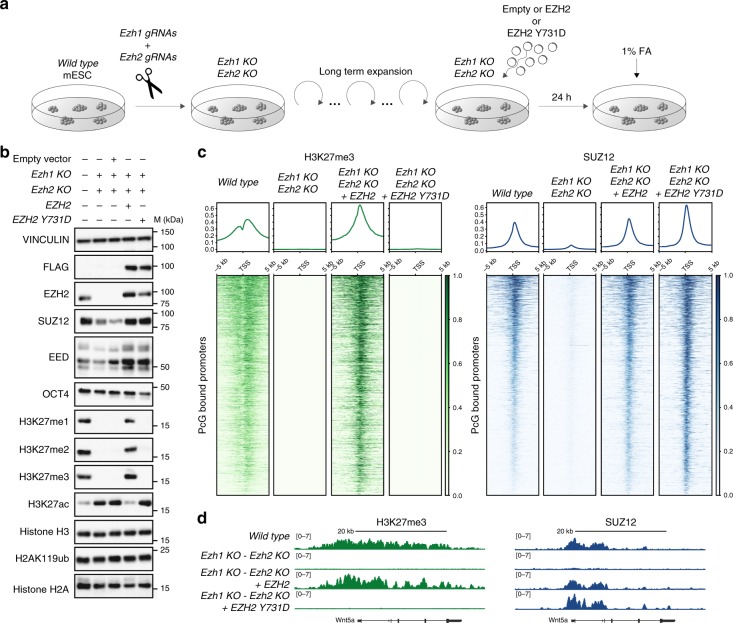
De novo PRC2 recruitment is independent of H3K27 methylation. **a** The experimental strategy used for de novo recruitment experiments. **b** Western blot analyses with the indicated antibodies using protein extracts obtained from WT, *Ezh1/2* dKO, and *Ezh1/2* dKOs cells transiently transfected with vectors expressing human EZH2 or EZH2Y731D (Y726D in mouse). Empty vector was used as negative control of transfection. Vinculin, histone H3, and histone H2A served as loading controls. **c** Heatmaps representing the spike-in normalized H3K27me3 ChIP-seq intensities and the normalized SUZ12 ChIP-seq signals ± 5 kb from TSS of SUZ12-bound promoters in the cell lines presented in **a**. Promoters were ranked according to SUZ12 intensities in WT mESCs. Upper panels, enrichment plots representing the average distribution of H3K27me3 and SUZ12 ± 5 kb around TSS. **d** Genomic snapshots for H3K27me3 and SUZ12 ChIP-seq analyses performed in the indicated cell lines at the *Wnt5a* locus

To further explore the properties of PRC2 recruitment to its target sites, and the role of H3K27me3 deposition in determining the specificity of this recruitment, we cultured *Ezh1/2* dKO ESCs in the absence of any form of PRC2 and H3K27 methylation for several weeks. We then transiently reintroduced the expression of a wild-type or Y731D mutant (equivalent to Y726D in mouse) of human EZH2 for 24 h (Fig. 3a). This window of expression was sufficient to restore not only SUZ12 and EED levels but also deposition of all forms of H3K27 methylation to normal (Fig. 3b). Moreover, consistent with results presented in Fig. 1b, the EZH2 Y731D mutation under these conditions was unable to recover H3K27 methylation (Fig. 3b). ChIP-seq analyses in the same cells confirmed these results and further demonstrated that PRC2 (SUZ12) can be recruited de novo to the same target sites independently of its ability to deposit H3K27me3 (Fig. 3c, d and Supplementary Fig. 6).

**Fig. 4:**
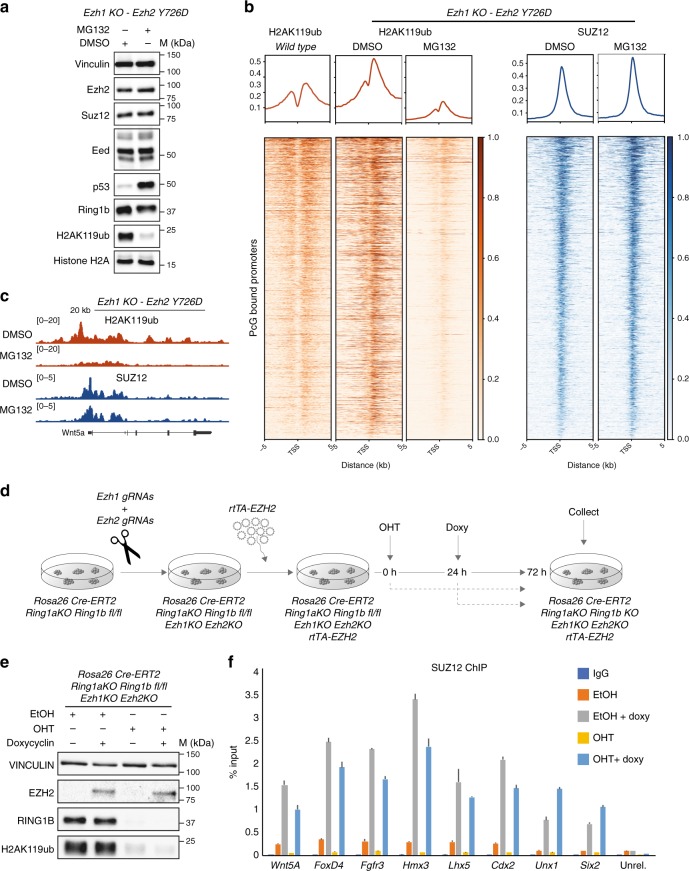
PRC2 recruitment is not affected by acute loss of H2Aub1 deposition. **a** Western blot analysis with the indicated antibodies of total protein extracts obtained from *Ezh1* KO-*Ezh2* Y726D cells upon treatment with DMSO (vehicle) or 10 µM MG132 for 6 h. TP53 served as positive control for MG132 treatment. Vinculin and histone H2A were used as loading controls. **b** Heatmaps representing the spike-in normalized H2AK119ub ChIP-seq intensities and the normalized SUZ12 ChIP-seq signals ± 5 kb around TSS of SUZ12-bound promoters/targets in *Ezh1* KO-*Ezh2* Y726D cells upon treatment with DMSO or MG132. Promoters were ranked according to their intensities in WT mESCs. Enrichment plots representing the average distribution of H2AK119ub and SUZ12 ± 5 kb around TSS are shown in the upper panels. **c** Genomic snapshot of H2AK119ub1 and SUZ12 of the analysis performed in **b** at the *Wnt5a* locus. **d** Experimental strategy used for de novo recruitment performed in Rosa26:Cre-ERT2, *Ring1a* KO, *Ring1b* fl/fl, *Ezh1* KO, *Ezh2* KO ESC transduced with a doxycycline inducible lentiviral vector expressing EZH2. **e** Western blot analysis with the indicated antibodies of total protein extracts obtained from cells described in **d**, treated with EtOH (vehicle) or OHT (0.5 μM) alone or in combination with doxycycline (1 μg/ml). Vinculin was used as loading control. **f** qPCR of SUZ12 ChIP in the same cells used in **e**, upon treatment with EtOH or OHT (0.5 μM) alone or in combination with doxycycline (1 μg/ml). Rabbit IgG served as negative control. Enrichments are normalized to % INPUT. Data are represented as mean ± SEM

PRC2 has been shown to have a specific binding affinity for H2Aub1 deposited by the PRC1 complex^32–34^. It is therefore possible that the ability of PRC2 to be recruited to PcG target sites in the absence of H3K27me3 could be mediated by the presence of H2Aub1, which was unchanged in *Ezh1/2* dKO and *Ezh2* Y726D ESC lines (Figs. 1b and 3b). As inactivation of RING1A/B activity induces a rapid loss of ESC viability^6^, we eliminated H2Aub1 with MG132 treatment, which rapidly reduces the pool of free ubiquitin in the cell (and thus also H2Aub1 levels)^42^. Indeed, after 6 h of MG132 treatment in *Ezh2* Y726D ESCs, H2Aub1 deposition was nearly abolished, but the global levels of core PRC2 components were not affected (Fig. 4a and Supplementary Fig. 7A, B). ChIP-seq analysis in these cells confirmed this result, demonstrating that H2Aub1 deposition is affected at all genomic sites that are also bound by the PRC1 complex in WT ESCs (Fig. 4b). Importantly, ChIP-seq analysis for SUZ12 in the same samples showed correct binding of the PRC2 complex at a genome-wide level, with no signs of displacement (Fig. 4b, c). To strengthen this conclusion, we generated *Ezh1/2* dKO in *Ring1A* KO ESC carrying a conditional allele for *Ring1B* (*Ring1b fl/fl*). We have further introduced in these cells a tetracycline inducible form of EZH2 to activate first, the loss of PRC1 activity by OHT treatment and second, the activation of EZH2 expression with doxycycline (doxy; Fig. 4d, e). ChIP analysis at PRC2 targets showed that also under these experimental conditions, SUZ12 was efficiently recruited at target sites regardless of PRC1 activity (Fig. 4f). This result was valid also for different PRC2 ancillary subunits (MTF2, JARID2 and PHF19) with the exception of EPOP, which seems to be affected in binding upon MG132 treatment (Supplementary Fig. 7C). Overall, these results suggest that the PRC2 complex is recruited to specific loci by intrinsic properties that are independent of its ability to bind either H3K27me3 or H2Aub1.

### EZH1 activity fully preserves PRC1-mediated repression

**Fig. 5:**
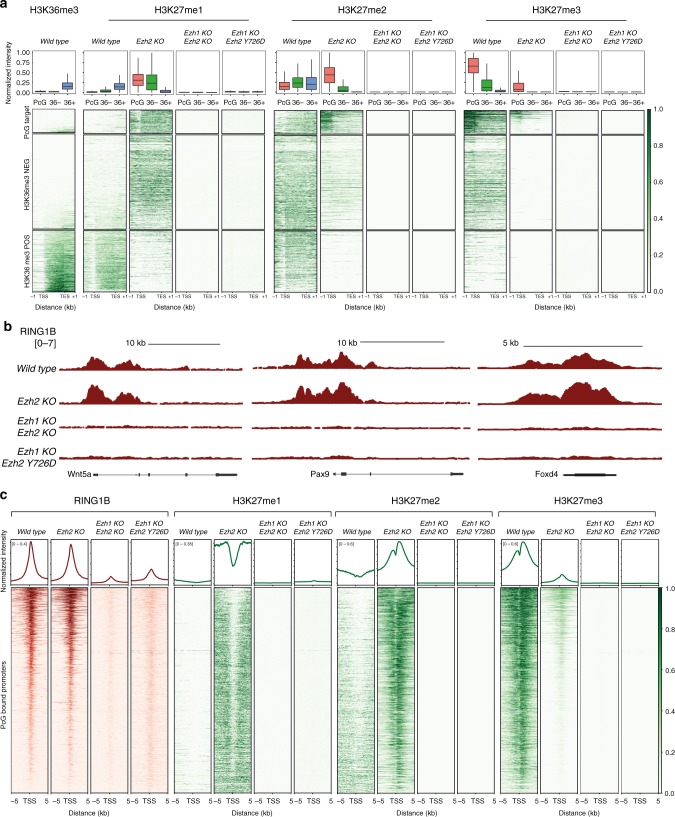
EZH1-mediated accumulation of H3K27me2 at promoters sustains maintenance of PRC1 repressive domains. **a** Heatmaps representing the spike-in normalized H3K36me3, H3K27me1, H3K27me2, and H3K27me3 ChIP-seq intensities within the entire normalized gene length plus a ± 1 kb region around all annotated RefSeq genes (*N* = 31027). Regions were clustered into Polycomb targets, H3K36me3 positive and negative regions. PcG targets were ranked for the intensity of H3K27me3 deposition while the rest of RefSeq genes for ascending intensity of H3K36me3 in WT cells. Upper boxplots represent the average normalized intensities of H3K36me3, H3K27me1, H3K27me2, and H3K27me3 in the indicated cell lines in the different gene clusters. **b** Representative genomic snapshots of RING1B ChIP-seq analyses performed in the indicated cell lines. **c** Heatmaps representing the normalized RING1B and the spike-in normalized H3K27me1, H3K27me2, and H3K27me3 ChIP-seq intensities ± 5 kb around TSS of Polycomb-bound promoters in the indicated cell lines. Promoters were ranked according to their intensities in WT mESCs. Enrichment plots representing the average distribution of RING1B, H3K27me1, H3K27me2, and H3K27me3 ± 5 kb around TSS are shown in the upper panels

Since loss of EZH2 strongly affected H3K27me2 deposition without altering H3K27me1 levels in bulk (Fig. 1b), we also profiled the deposition of these two PRC2-dependent modifications by ChIP-seq analyses. We stratified all RefSeq genes in three groups: PcG targets, non-PcG targets with low H3K36me3 levels and non-PcG targets with high H3K36me3 deposition (Fig. 5a). Consistent with Fig. 1b results, *Ezh2* KO, *Ezh1/2* dKO and *Ezh2* Y726D ESC showed a complete loss of H3K27me2 deposition at the intragenic regions of poorly expressed genes. However, in *Ezh2* KO ESC, H3K27me2 accumulated at PcG bound promoters compensating the reduction in H3K27me3 (Fig. 5a). Similarly, H3K27me1 accumulated at poorly transcribed intragenic regions where H3K27me2 was lost. Unexpectedly, H3K27me1 deposition at highly transcribed genes was completely lost in *Ezh2* KO ESC, suggesting that EZH2 could be specifically involved in supporting this activity (Fig. 5a).

We took advantage of these mutants to further explore the relationship between PRC1 and PRC2 recruitment. We first analyzed RING1B chromatin association by ChIP-seq analyses in WT, *Ezh2* KO, *Ezh1/2* dKO, and *Ezh2* Y726D ESCs. As expected, RING1B was efficiently associated to the same repertoire of genomic loci bound by PRC2 in WT ESCs, confirming that both complexes co-occupy the vast majority of target sites (Supplementary Figs. 2B, 5B, and 5C). Importantly, RING1B binding was completely abolished in both *Ezh1/2* dKO and *Ezh2* Y726D ESCs, demonstrating that RING1B is recruited to PcG target loci by H3K27me3 deposition independently of whether or not PRC2 is associated to the same site (Fig. 5b, c). However, RING1B was not displaced in *Ezh2* KO ESCs retaining an unaltered chromatin association profile identical to WT ESCs. Consistent with the good affinity of CBX7 in binding also H3K27me2^43^, these results showed that the remaining H3K27me3 together with H3K27me2 accumulation is sufficient to preserve efficient PRC1 recruitment to chromatin at PcG sites in the absence of EZH2 activity.

### H3K27ac plays an active role in cell identity control

**Fig. 6:**
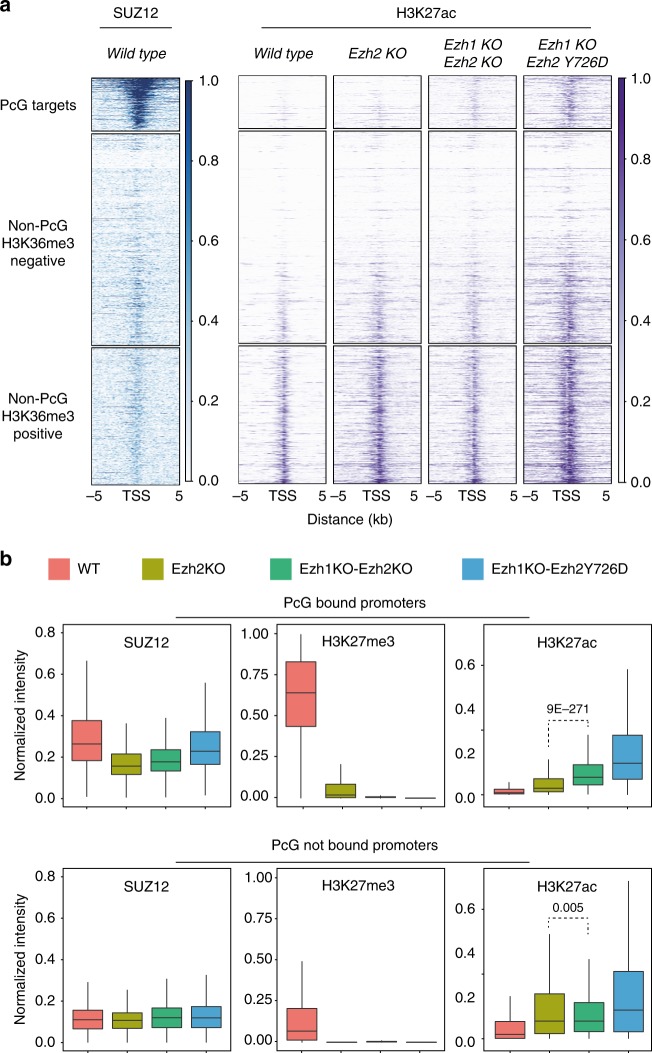
H3K27me2/3 deposition, but not PRC2 occupancy, prevents H3K27ac invasion at PcG repressed promoters. **a** Heatmaps representing the normalized SUZ12 and the spike-in normalized H3K27ac ChIP-seq signals ± 5 kb around all TSS annotated in RefSeq. Promoters were clustered into Polycomb targets, H3K36me3 positive and H3K36me3 negative. PcG targets were ranked for the intensity of H3K27me3 deposition while the rest of RefSeq genes for ascending intensity of H3K36me3. **b** Boxplots representing the normalized intensities of SUZ12 and the spike-in normalized intensities of H3K27me3 and H3K27ac in the indicated cell lines at Polycomb-bound promoters (upper panels) and at Polycomb not-bound promoters (bottom panels). *P*-values were determined by Wilcoxon test

Consistent with previous reports^39,40^, loss of PRC2 activity resulted in a global increase of H3K27ac levels (Fig. 1b). Although such increases occur sparsely along the genome as a consequence of H3K27me2 loss, H3K27ac preferentially accumulates both at promoters and distal regulatory sites^17,44^. Focusing on all RefSeq genes transcription start sites (TSS) in WT ESCs, we observed that H3K27me3 and H3K27ac were deposited in a mutually exclusive manner, with PcG target promoters showing undetectable H3K27ac levels, while active promoters showing accumulation (Fig. 6a, b). Upon loss of PRC2 activity (in *Ezh1/2* dKOs), the levels of H3K27ac increased globally, invading promoters that were previously decorated by H3K27me3 (Fig. 6a, b). H3K27ac promoter invasion also occurred in *Ezh2* Y726D ESCs, even though these cells have a catalytically inactive PRC2 that remained bound at these sites. Importantly, H3K27ac invasion at these promoters was significantly prevented in *Ezh2* KO ESC by residual H3K27me3 deposition and H3K27me2 accumulation (Fig. 6a, b). Consistent with previous reports^45^, accumulation of H3K27ac did not result in differential chromatin accessibility at both promoters and enhancer sites (Supplementary Fig. 8A–C) and was not a consequence of a generally increased P300 and CBP activity (Supplementary Fig. 9A, B). Interestingly, while acetylation of other lysine residues of the H3 n-terminal tail was unaffected, some residues of histone H4 also gained acetylation in a PRC2-dependent manner (Supplementary Fig. 9C), suggesting an unexpected crosstalk. Overall, these results demonstrated that the major determinant for restricting acetylation at promoters is the methylated status of H3K27, regardless of PRC2 chromatin association.

**Fig. 7:**
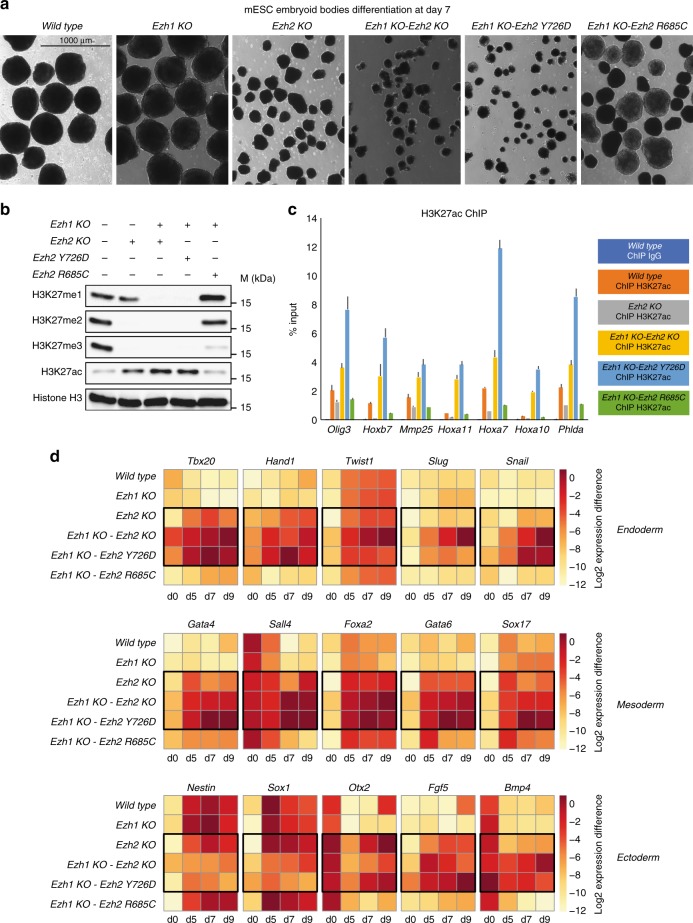
Diffused accumulation of H3K27ac directly contributes to PRC2-mediated differentiation defects. **a** Pictures of embryoid bodies at day 7 (d7) of differentiation, from the indicated ESC lines (shown at ×4 magnification). **b** Western blot analysis using the indicated antibodies with protein extracts obtained from WT, *Ezh2* KO, *Ezh1/2* dKO, *Ezh1* KO-*Ezh2* Y726D, and *Ezh1* KO-*Ezh2* R685C mouse ESC lines. Histone H3 was used as loading control. **c** ChIP-qPCR of H3K27ac in the indicated mESC lines. IgG rabbit served as negative control. Enrichments are normalized to % INPUT. Data are represented as mean ± SEM. **d** Heatmaps of qPCR relative expression analyses for the indicated genes at d0, d5, d7, and d9 during ESC differentiation of the cells indicated shown in **a**. *Gapdh* expression served as a normalization control

To investigate how distinct chromatin states contribute to the differentiation capabilities of ESCs, we tested whether the different mutants could form embryoid bodies (EBs) in cell culture. Consistent with the complete compensation for all H3K27 methylation states exerted by EZH2, *Ezh1* KO EBs displayed normal morphology with respect to WT cells (Fig. 7a). In contrast, *Ezh2* KO*, Ezh1/2* dKO, and *Ezh2* Y726D mutant ESCs displayed compromised differentiation capabilities (Fig. 7a). This highlights that maintenance of repressive domains at target promoters by H3K27me2/3 and PRC1 recruitment is not sufficient to prevent developmental failure, suggesting a potential role for diffused chromatin H3K27 hyperacetylation in PRC2 developmental defects. To gain further evidence in this direction, we generated in an *Ezh1* KO background a new EZH2 mutation (*Ezh2* R685C; Supplementary Fig. 10A) that showed a preferential rescue of H3K27me2 activity respect to H3K27me3 (Fig. 7b). This resulted in attenuated bulk increase of H3K27ac (Fig. 7b) respect to *Ezh2* KO, with no spreading at PRC2 target promoters (Fig. 7c). Importantly, *Ezh2* R685C EBs showed a morphology that look more similar to *wild-type* ESC (Fig. 7a). While all mutant ESC expressed and downregulated pluripotency markers when differentiated in EBs (Supplementary Fig. 10B), *Ezh2* KO, *Ezh1/2* dKO, and *Ezh2* Y726D EBs failed to correctly activate markers of differentiation for different lineages (Fig. 7d). In contrast, these defects were markedly attenuated in the *Ezh2* R685C EBs in agreement with their normal morphology (Fig. 7a, d). These results demonstrated that establishment of H3K27me3/2 deposition at target promoters and maintenance of PRC1 repressive domains is not sufficient to prevent developmental failure (Fig. 7a). These data further suggest that a diffused increase in chromatin H3K27ac could play a direct role in PRC2-dependent loss of cell identity control.

## Discussion

Here, we have demonstrated that, in absence of PRC2-EZH2 activity, PRC2-EZH1 is only able to sustain H3K27me1 deposition at a global level in vivo. Using more sensitive ChIP-seq analyses, we discerned that H3K27me3 is nonetheless still significantly deposited at all PRC2 target promoters ( > 70%) identified in WT ESCs, and compensated by H3K27me2 (Fig. 5). Thus, although with a reduced efficiency, PRC2-EZH1 retained the ability to target and modify the same repertoire of target promoters of PRC2-EZH2 in ESCs. This is in line with the functional and biochemical proprieties of PRC2-EZH1 and PRC2-EZH2 shown by several studies. In general, PRC2-EZH2 is primarily linked to cell proliferation, and PRC2-EZH1, to differentiated post-mitotic conditions^19,46^. A series of biochemical studies revealed the importance of allosteric stimulation of PRC2 for the nucleation and spreading of H3K27me3 domains at a genome-wide level, demonstrating its importance in sustaining H3K27me2/me3 deposition^47^. Notably, minor differences in the amino acid sequence of the stimulatory responsive domain between EZH1 and EZH2 render EZH1 less responsive to allosteric stimulation than EZH2^48^. Importantly, PRC2-EZH1-mediated H3K27me2/3 deposition at target genes was sufficient to preserve PRC1 binding and gene repression (Figs. 2e and 5b, c).

The mechanisms by which PRC2 is targeted to chromatin play an important aspect of PRC2 activity regulation. Different biochemical features can stabilize PRC2 recruitment to target sites, including its intrinsic affinity for CpG-rich DNA elements^29,49,50^, long non-coding RNAs^51–53^, and ancillary proteins associated to PRC2^31^. Recent work has further identified specific CG-rich consensuses that seem to serve as nucleation sites for PRC2 recruitment and spreading^47^. Different forms of PRC2 also have distinct affinities in binding chromatin templates. For instance, EZH1 was shown in vitro to bind nucleosome templates with a greater affinity than EZH2^48^. Moreover, different analyses studying the function of ancillary proteins, such as MTF2, JARID2, and AEBP2, have shown that all of these activities contribute to stabilizing PRC2 at chromatin and to stimulating its in vitro activity^23,34,54^. In particular, JARID2 can be methylated by EZH2 on K116, resulting in allosteric stimulation with the same mechanism as for H3K27me3^55^. While JARID2 plays a crucial role in the context of ESCs, its function in more-committed cell types is marginal, suggesting that different mechanisms for stimulatory actions and recruitment exist. Recent work from the Helin laboratory has further shown that a form of SUZ12 unable to associate with PRC2 is still recruited to PcG target sites^41^. Our results in *Ezh1/2* dKO ESC also show that SUZ12 binding is retained at PcG sites, however with lower intensity respect to *wild-type* ESC. The catalytically inactive form of PRC2 supports this notion, showing that PRC2 can associate to all its target sites at a genome-wide level in the absence of any form of H3K27 methylation (Fig. 1). In these conditions, SUZ12 binding was stabilized respect to *Ezh1/2* dKO cells, suggesting SUZ12 assembly into a PRC2 complex favors its chromatin association independently of PRC2 catalytic activity.

How PRC2 acquires specificity for target gene recognition in specific cell lines is also an important open question. Here, we showed that in ESCs that had been cultured for an indefinite number of passages in the absence of any form of PRC2 activity (*Ezh1/2 d*KO), H3K27me3 deposition and PRC2 target association were fully restored within 24 h after EZH2 re-expression (Fig. 3) confirming that specificity remains an intrinsic feature of cellular identity. PRC2 is attracted to CpG-rich DNA elements^49^, an affinity linked to MTF2 DNA binding proprieties^26^, and to specific motifs within CpG islands (CpGi) that were linked to PRC2 de novo nucleation^47^. The lack of transcription from CpG-rich promoters was further proposed as a determinant for PRC2 recruitment, which would imply that all CpGi are potential targets of PRC2^30^. Importantly, our catalytically inactive EZH2 mutant was recruited to all PRC2 target sites, despite its inability to deposit any form of H3K27 methylation. Although in agreement with the H3K27me3 nucleation and spreading model^47^, this result further suggests that de novo binding of PRC2 to all target sites is uncoupled from H3K27me3 nucleation. Overall, our data link the models of PRC2 being tethered by transcriptional silencing with the mechanisms of spreading H3K27me3 domains.

PRC2 was also shown to retain biochemical affinities for H2Aub, which is deposited by PRC1 activity. Further, loss of RING1A/B activity can affect the association of PRC2 at target sites in ESCs, suggesting that H2Aub1 is a determinant for specifying PRC2 target association^32–34^. However, in *Ezh2* Y726D ESCs, H2Aub1 levels were not affected either at bulk levels (Fig. 1b) or in genome-wide deposition (Fig. 4b). This suggests that retained H2Aub1 deposition could serve as a docking site for de novo PRC2 recruitment and H3K27me3 deposition, thus defining genome-wide binding specificity. Our results now clearly show that acute depletion of H2Aub1 neither results in release of PRC2 nor affects PRC2 ability to be re-recruited at is target loci, regardless of the presence of any form of H3K27 methylation (Fig. 4). Based on this result, we would suggest that PRC2 binding affinity for H2Aub1 serves more to reinforce already-formed PcG repressive domains than to define de novo sites.

Our data further revealed that PRC2-EZH1 was still able to deposit H3K27me3 at virtually all PRC2 target sites that we classified in WT ESCs. A careful analysis of this activity in *Ezh2* KO ESCs highlighted that H3K27me3 deposited by EZH1 was confined within the boundaries of the association of PRC2 with chromatin and was unable to spread outside these regions (Fig. 2). This suggests that an inefficient allosteric stimulation previously measured in vitro for PRC2-EZH1^48^ is likely to confine H3K27me3 deposition within the boundaries of PRC2 physical association with chromatin. Nevertheless, within these boundaries, PRC2-EZH1 was able to deposit H3K27me3 at levels comparable to those in WT ESCs (Fig. 2), further accumulating H3K27me2 (Fig. 5a). Interestingly, EZH1 accumulated H3K27me2 at these sites but failed to normally deposit H3K27me1 at transcribed genes, suggesting specific contributions of the two methyltransferases in defining the H3K27 methylation landscape. Overall, our data as well as those from others^41,47,48,56^ demonstrate that PRC2 recruitment is uncoupled from the mechanism by which H3K27me3 repressive domains are formed, highlighting the importance of allosteric EZH2 stimulation for spreading but not for deposition of H3K27me3 at all PcG target sites.

In *Drosophila melanogaster*, PcG proteins are recruited to target sites via the recognition by the DNA binding transcription factors of DNA elements, named PREs, which can be located at considerable distances from target promoters^27^. This results in the establishment of very large H3K27me3 domains with respect to what is observed in mammalian genomes^57^. This may suggest that repressive mechanisms have diverged to adapt to the changes in the evolution of distinct genomic features. Work of the Muller laboratory has demonstrated that fully penetrant H3K27 amino acid substitutions can phenocopy the homeotic transformations observed for PRC2 mutants during fly development^37^. This points at H3K27me3 as an essential executor of developmental functions, but whether this is true also for mammals remained unclear. Taking advantage of our catalytically inactive PRC2 mutant ESCs, we have now demonstrated that the absence of H3K27 methylation, but not of PRC2 association to target promoters per se, phenocopied the developmental defects observed in *Ezh1/2* dKO developing EBs (Fig. 7). This demonstrates that deposition of H3K27 methylations, but not PRC2 recruitment to target sites, is an essential feature for at least early developmental decisions.

It is important to highlight that developmental defects very similar to those in catalytically inactive PRC2 mutant ESC lines were also observed in *Ezh2* KOs but not in *Ezh1* KOs. The lack of such defects in *Ezh1* KOs further confirms the essential role of H3K27 methylation, as EZH2 fully compensated loss of EZH1 activity (Fig. 1). In contrast, the defects observed for *Ezh2* KOs highlights that an altered deposition of H3K27 methylated forms is critical in early developmental decisions. Both results are in complete agreement with the lack of developmental defects previously observed in *Ezh1* KO mice^20^ and with the early embryonic lethality reported for *Ezh2* KO embryos during gastrulation stages^2^. These developmental defects were phenocopied by complete loss of PRC2 activity in *Eed* or *Suz12* KO embryos^3,4^.

One critical activity of PRC2 is its ability to affect PRC1 recruitment to chromatin. PRC1 can exist in different forms that can be roughly divided in canonical and non-canonical forms. Canonical PRC1 complexes are characterized by the presence of CBX proteins, which retain binding affinities for H3K27me3 substrates^31^. Our results clearly demonstrate that RING1B was severely displaced in the absence of H3K27me3 deposition at target sites, regardless of the presence or absence of PRC2 at target promoters (*Ezh1/2* dKO vs. *Ezh2* Y726D). This accounts for approximately 90% of total RING1B associated at PcG sites in agreement with previous reports using *Eed* KO ESCs^58^. The massive RING1B displacement (Fig. 5b, c) further suggests that canonical PRC1 remains the predominant form active in ESCs and that its chromatin displacement cannot be fully compensated by additional recruitment of non-canonical forms. Our data further demonstrated that RING1B binding was fully retained at target sites in the absence of PRC2-EZH2 activity, likely by the localized deposition of H3K27me3 and accumulation of H3K27me2 by PRC2-EZH1 (Fig. 5). Indeed, CBX7 still retains a significant affinity in binding H3K27me2 respect to H3K27me3 (Kd: ~ 136 vs. ~ 22 µM µM, respectively^43^). Importantly, while H3K27me2/3 deposition and PRC1 association at target sites were sufficient to maintain target genes repression, they were not sufficient to prevent developmental failure when *Ezh2* KO ESCs were challenged to differentiate into EBs. This suggests that additional features to the direct control of target genes repression could contribute to proper development. Our data using the *Ezh2* R685C mutant further suggest that diffused chromatin H3K27ac could be a major determinant in PRC2-mediated developmental defects.

We have previously shown that the distinct forms of H3K27 methylation are spatially organized along the genome. H3K27me2 is broadly diffused and modifies >70% of histone H3 present in ESCs^17^. In addition, we and others have shown that loss of PRC2 enzymatic activity always results in an aberrant increase in the levels of H3K27 acetylation^39,40^. We have previously mapped the sites of H3K27ac accumulation in *Eed* KO ESCs and have shown that such deposition occurs at both H3K27me3-decorated promoters and in the intra- and intergenic space at which H3K27me2 is broadly diffused^17^. As this can result in an altered activation of enhancer elements, we speculate that loss of H3K27me2 deposition could have a major role in PRC2-dependent deregulated control of cell identity. Although *Ezh2* KO ESCs showed a similar gain in H3K27ac as *Ezh1/2 d*KO and *Ezh2* Y726D ESCs, our ChIP-seq analyses for H3K27ac in *Ezh2* KOs demonstrated that all PcG target promoters were more protected from H3K27 hyperacetylation (Fig. 6). This suggests that in the absence of EZH2 activity, PcG target promoters remain protected by the presence of local H3K27me3 deposition, accumulation of H3K27me2 and maintenance of PRC1 binding. This may link diffused H3K27 hyperacetylation with developmental failure. Indeed, this is further supported by the attenuated differentiation defects observed in the *Ezh2* R685C mutant, which correlate with their reduced accumulation of H3K27ac.

Overall, our results provide evidences to suggest that diffused chromatin hyperacetylation could directly contribute to the early developmental defects observed in PRC2 defective ESCs. In agreement with previous reports, our data show that this does not involve genome-wide changes in chromatin accessible sites. Whether this involves a specific unbalance in enhancer activity or a more global effect on chromatin three-dimensional conformation are interesting open questions to pursue. In light of the specific roles that PRC1 and PRC2 play in distinct adult tissues^20,59–63^, it will now also be very important to understand the contributions of diffuse H3K27 hyperacetylation in the homeostasis of organs and tissues. Increased H3K27 acetylation in DIPG pediatric brain tumors, in which PRC2 activity is inhibited by the expression of a H3K27M mutation, renders tumors cells more sensitive to BET inhibitors^64^. Therefore, considering the frequent gain- and loss-of-function mutations that affect different PRC2 subunits, it will be important to explore the contributions of these mechanistic aspects in different pathological contexts.

## Methods

### Cell lines and cell culture

ESCs KO clones were generated in E14TG2a by CRISPR/Cas9 targeting *Ezh1* or *Ezh2* genes using double sgRNAs to excise the whole gene of interest. Clones were screened by PCR followed by Sanger sequencing to confirm the macrodeletions. *Ezh2* Y726D and *Ezh2* R685C ESCs were obtained by precise editing using a specific single-stranded template (ssODN, IDT) in *Ezh1* KO cells. Introduction of the desired point mutation was confirmed by restriction analysis followed by Sanger sequencing.

mESCs were grown on 0.1% gelatin-coated dishes in 2i/LIF-containing GMEM medium (Euroclone) supplemented with 20% fetal calf serum (Euroclone), 2 mM glutamine (Gibco), 100 U/ml penicillin, 0.1 mg/ml streptomycin (Gibco), 0.1 mM non-essential amino acids (Gibco), 1 mM sodium pyruvate (Gibco), 50 µM ß-mercaptoethanol phosphate-buffered saline (PBS; Gibco), 1000 U/ml leukemia inhibitory factor (LIF; produced in-house), and GSK3β and MEK 1/2 inhibitors (ABCR GmbH) to a final concentration of 3 μM and 1 μM, respectively.

Rosa26:Cre-ERT2 *Ring1A* KO *Ring1B* fl/fl conditional mESC and *Ring1b* KO mESC lines were generated elsewhere^6,9^. Ring*1A*-/-; *Ring1B* fl/fl;Rosa26::CreERT2 conditional mESC were subjected to CRISPR/Cas9 editing in order to obtain double *Ezh1/Ezh2* KO. Cells were transduced with 5 μg/ml polybrene and lentiviral particles delivering pLIX_402 vector (Addgene #41394) expressing human wild-type EZH2 for 36 h and then subjected to 72 h -long puromycin-selection (1 μg/ml). For inducible EZH2 re-expression, cells were treated for 24 h with 0.5 μM 4-hydroxytamoxifen (4-OHT; or EtOH as control) in order to obtain *Ring1b* KO; then were treated with a combination of 4-OHT (or EtOH) and doxycycline (1 μg/ml) for 48 h prior harvesting.

mESCs were induced to differentiate to EBs by LIF removal in hanging drops (1000 cells/20 μl drop) on the lid of 15 cm petri dishes for 48 h. EBs were then collected and stimulated with 0.5 µM all-trans retinoic acid (ATRA) for 3 days. EBs were left in culture in non-coated petri dishes in ES medium without LIF until day 7. Medium was replaced every second day.

Where indicated, undifferentiated mESCs were treated with 10 μM MG132 (Calbiochem Merck) dissolved in 100% DMSO for 6 h.

### Transfections

For rescue experiments, pCAG vectors encoding Flag-Avi-tagged human wild-type EZH2 or EZH2 Y731D mutant or pCAG vector encoding Flag-HA-tagged human wild-type EZH1 were transfected into mESCs using Lipofectamine 2000 (ThermoFisher Scientific), according to the manufacturer’s instructions. Cells were subjected to puromycin selection (2 µg/ml) for 24 h and then collected for subsequent analyses.

### Western blot

For western blot analysis, mESCs were lysed and sonicated in ice-cold S300 buffer (20 mM Tris-HCl pH 8.0, 300 mM NaCl, 10% glycerol, 0.2% NP-40) and supplemented with protease inhibitors (Roche). For co-immunoprecipitation experiments, cells were lysed in the same lysis buffer. Immunoprecipitation was performed on 1 mg protein extracts using homemade anti-EZH2 (AC22) cross-linked sepharose beads (30 μl slurry for IP, Healthcare, cat. # 170780-01) for 3 to 4 h at 4 °C. Immunocomplexes were washed 5× with S300 buffer and eluted in Laemmli sample buffer. Protein lysates were separated on sodium dodecyl sulfate polyacrylamide gel electrophoresis gels and transferred to nitrocellulose membranes. After probing with the suitable primary and secondary antibodies, chemoluminescence signals were captured with the ChemiDoc Imaging System (Bio-Rad).

### Antibodies

Western blot analyses were performed with: anti-Vinculin (1:8000 dilution; V9131; Sigma-Aldrich), anti-Oct3/4 (1:1000 dilution; sc5279; Santa Cruz Biotechnology), anti-Ezh2 (1:10 dilution; BD43 clone; homemade^3^), anti-Suz12 (1:1000 dilution; sc-46264; Santa Cruz Biotechnology), anti-Eed (1:10 dilution; AA19 clone; homemade^65^), anti-p53 (1:5 dilution: homemade), anti-flag (1:1000 dilution; F3165; Sigma-Aldrich), anti-Mtf2 (1:1000 dilution; 16208-1-AP; Proteintech), anti-Jarid2 (1:1000 dilution; ab48137; Abcam), anti-Phf19 (1:1000 dilution; 11895-1-AP; Proteintech), anti-EPOP (1:1000 dilution)^66^, anti-P300 (1:500 dilution; sc-585; Santa Cruz Biotechnology), anti-CBP (1:200 dilution; sc-583; Santa Cruz Biotechnology), anti-HA (1:10 dilution; 12CA5 clone; homemade), anti-H3K27me1 (1:1000 dilution; 61015; Active Motif), anti-H3K27me2 (1:1000 dilution; 9728; Cell Signaling Technology), anti-H3K27me3 (1:1000 dilution; 9733; Cell Signaling Technology), anti-H3K27ac (1:1000 dilution; ab4729; Abcam), anti-H3K9K14ac (1:1000 dilution; C15410200; Diagenode), anti-H3K14ac (1:500 dilution; 39599; Active Motif), anti-H3K18ac (1:500 dilution; E-AB-20285; Microtech), anti-H3K23ac (1:500 dilution; E-AB-20205; Microtech), anti-H4K5ac (1:1000 dilution; 39170; Active Motif), anti-H4K8ac (1:500 dilution; E-AB-20208; Microtech), anti-H4K12ac (1:500 dilution; 39166; Active Motif), anti-H4 (1:1000 dilution; ab7311; Abcam), anti-H2AK119ub (1:1000 dilution; 8240; Cell Signaling Technology), anti-H3 (1:8000 dilution; 1791; Abcam), and anti-H2A (1:1000 dilution; 12349; Cell Signaling Technology).

ChIP assays were performed using: anti-Suz12 (3737; Cell Signaling Technology), anti-Ring1b (homemade;^63^), anti-HA (12CA5 clone; homemade), anti-Jarid2 (ab48137; Abcam), anti-Mtf2 (16208-1-AP; Proteintech), anti-Phf19^67^, anti-EPOP^66^, anti-H3K27me1 (61015; Active Motif), anti-H3K27me2 (9728; Cell Signaling Technology, anti-H3K27me3 (9733; Cell Signaling Technology), anti-H3K27ac (ab4729; Abcam), anti-H3K36me3 (4909; Cell Signaling Technology), anti-H2AK119ub (8240; Cell Signaling Technology) and purified rabbit IgG (I5006; Sigma-Aldrich).

### Quantitative real-time PCR (qPCR)

Total RNA was extracted with the Quick-RNA™ MiniPrep extraction kit (Zymo Research) and retro-transcribed with ImProm-II™ Reverse Transcription System (Promega) according to the manufacturer’s instructions. Quantitative real-time PCR (qPCR) was carried out using GoTaq qPCR master mix (Promega) on CFX96 Real-Time PCR Detection System (Bio-Rad). *Gapdh* was used as a control gene for normalization.

Graphical representations of qPCR analysis were generated in *R* by function “pheatmap”. Primer sequences are available upon request.

### RNA-seq

RNA-seq was performed following SMART-seq2 protocol^68^ with minor modifications. Briefly, poly-A containing mRNA molecules obtained from 1 μg of total RNA were copied into first-strand cDNA by reverse transcription and template-switching using oligo(dT) primers and an LNA-containing template-switching oligo (TSO). Resulting cDNA was pre-amplified with KAPA HotStart Taq enzyme (Kapa Biosystems) and then purified with Ampure beads (Agencourt AMPure XP- Beckman Coulter). Two nanograms of pre-amplified cDNA were tagmented with in-house produced Tn5 transposase and further amplified with KAPA HotStart Taq enzyme. After purification with Ampure beads, the quality of the obtained library was assessed by Bioanalyzer (High Sensitivity DNA kit, Agilent Technologies), prior sequencing.

### RNA-seq analysis

Reads were aligned to the mouse reference genome mm9 using TopHat v2.1.1^69^. Duplicates were removed using PICARD (http://broadinstitute.github.io/picard/). HTseq-count v0.8.0^70^ was used to calculate counts with parameters --stranded = no --mode = intersection-nonempty using RefSeq mm9 annotation downloaded from UCSC. DESeq2 v1.20 R package was used to perform differential expression analyses using default parameters^71^. Genes with an absolute log2 fold change of 1 were considered as differentially expressed.

### Chromatin immunoprecipitation (ChIP)

ChIP experiments were performed according to standard protocols^7^. Briefly, 1% formaldehyde cross-linked chromatin was sheared to 500–1000 bp fragments by sonication and incubated overnight in IP buffer (33 mM Tris-HCl pH 8, 100 mM NaCl, 5 mM EDTA, 0.2% NaN_3_, 0.33% SDS, 1.66% Triton X-100) at 4 °C with the indicated antibodies. For ChIP for SUZ12 and RING1B, 400–500 μg of chromatin and 5 μg of antibodies were used. For ChIP for histone modifications, 200–250 μg of chromatin supplemented with 5% spike-in of S2 Drosophila chromatin (prepared in the same manner) and 3 μg of antibodies were used. The next day, chromatin lysates were incubated for 2 to 4 h with protein-A sepharose beads (GE Healthcare). Beads were washed 3× with low-salt buffer (150 mM NaCl, 20 mM Tris-HCl pH 8, 2 mM EDTA, 0.1% SDS, 1 % Triton X-100) and 1× with high-salt buffer (500 mM NaCl, 20 mM Tris-HCl pH 8, 2 mM EDTA, 0.1% SDS, 1% Triton X-100), and then re-suspended in de-crosslinking solution (0.1 M NaHCO3, 1% SDS). DNA was purified with QIAquick PCR purification kit (Qiagen) according to the manufacturer’s instructions. DNA libraries were prepared with 2–10 ng of DNA using an in-house protocol^72^ by the IEO genomic facility and sequenced on an Illumina HiSeq 2000.

### ChIP-seq data analysis

Sequencing reads were aligned to the mouse reference genome (mm9) using Bowtie^73^, favoring only unique alignments and filtering out duplicates. For ChIP-RX experiments, sequencing reads were also aligned to the Drosophila reference genome (dm6) and used to compute a normalization factor for individual datasets. Peak calling was performed from aligned reads using MACS2^74^. Significant bound regions were defined using a cut-off of *p* = 1 × 10^–1^. BigWig files were built from Bam files with 200 bp reads, extension, input subtraction, and library size scaling using the function bamCompare from deepTools 2.0^75^. For ChIP-Rx samples the scaling factor was calculated as described in ref. ^76^. Heatmaps were generated using the computeMatrix and plotHeatmap utilities from deepTools^75^. The matrix was normalized by a factor calculated as 1/(max – min) when samples were subjected to ChIP analysis with the same antibody. The reference point for plotting was selected with respect to the transcription start site (TSS), and the 5000 bp distances upstream and downstream of TSS (TSS ± 5 kb) were graphically represented. Tracks were subsequently visualized using the IGB genome browser^77^.

Boxplots were generated with the package ‘ggplot’’ of *R*. H3K27me3 density distribution was computed within SUZ12 peaks and within a 1.2 kb region of 5′ and 3′ of SUZ12 peak boundaries. Target regions (*N* = 3968) were selected considering SUZ12 peaks colocalizing with RING1B with *P* ≥ 1 × 10^–1^ in wild-type mESCs. Correlation analysis was performed between wild-type and *Ezh2* KO samples. The IN/OUT density ratio was plotted, and linear regression analysis was calculated in *R*. *T*-test statistics were applied.

### ATAC-Seq

ATAC-seq was performed following previous methods^78^ with minor modifications. Briefly, 50,000 mESCs were directly lysed (lysis buffer: 10 mM Tris-HCl pH 7.4, 10 mM NaCl, 3 mM MgCl_2_, 0.1% NP-40), re-suspended in 50 μl of TAPS-buffer 1 × (TAPS 5x buffer: 50 mM TAPS-buffer, pH 8.3 and 25 mM MgCl_2_) added with 8% PEG and in-house produced Tn5 transposase and incubated at 37 °C for 1 h at slow agiting (500 rpm). After a double purification with Ampure beads (Agencourt AMPure XP- Beckman Coulter) recovered tagmented DNA was amplified and barcoded by PCR performed with KAPA HotStart Taq enzyme (Kapa Biosystems).

After size selection step with Ampure beads, libraries were eluted in 22 μl of pure water, quantified with Qubit fluorometer (Invitrogen) and checked with Bioanalyzer instrument (High Sensitivity DNA kit, Agilent Technologies), prior sequencing.

### ATAC-seq data analysis

Paired sequencing reads were aligned to the mouse reference genome (mm9) using Bowtie v1.2.2^73^, favoring only unique alignments and filtering out duplicates with PICARD. Heatmaps were generated using the computeMatrix and plotHeatmap utilities from deepTools^75^. Boxplot of normalized intensity was performed with ggplot. Enhancers annotation was obtained from previous work in ESC^79^.

### Reporting summary

Further information on experimental design is available in the Nature Research Reporting Summary linked to this article.

## Supplementary information

Supplementary Information

Reporting Summary

Source Data

## Data Availability

All relevant data supporting the key findings of this study are available within the article and its Supplementary Information files or from the corresponding author upon reasonable request. All ChIP-seq, RNA-seq, and ATAC-seq datasets are available at GEO under the accession number GSE116603. A source data file is provided as a Supplementary Dataset. A reporting summary for this Article is available as a Supplementary Information file.
