# Supplementary material for: Dissecting the role of H3K27 acetylation and methylation in PRC2 mediated control of cellular identity
Source: Nat Commun. 2019 Apr 11;10:1679. doi: 10.1038/s41467-019-09624-w (PMC6459869; doi:10.1038/s41467-019-09624-w)
Supplement: Supplementary file 1 — Supplementary Information [file 41467_2019_9624_MOESM1_ESM.pdf]

Supplementary Information

Dissecting the Role of H3K27 Acetylation and Methylation in PRC2 Mediated Control of Cellular Identity

Lavarone *et al*.

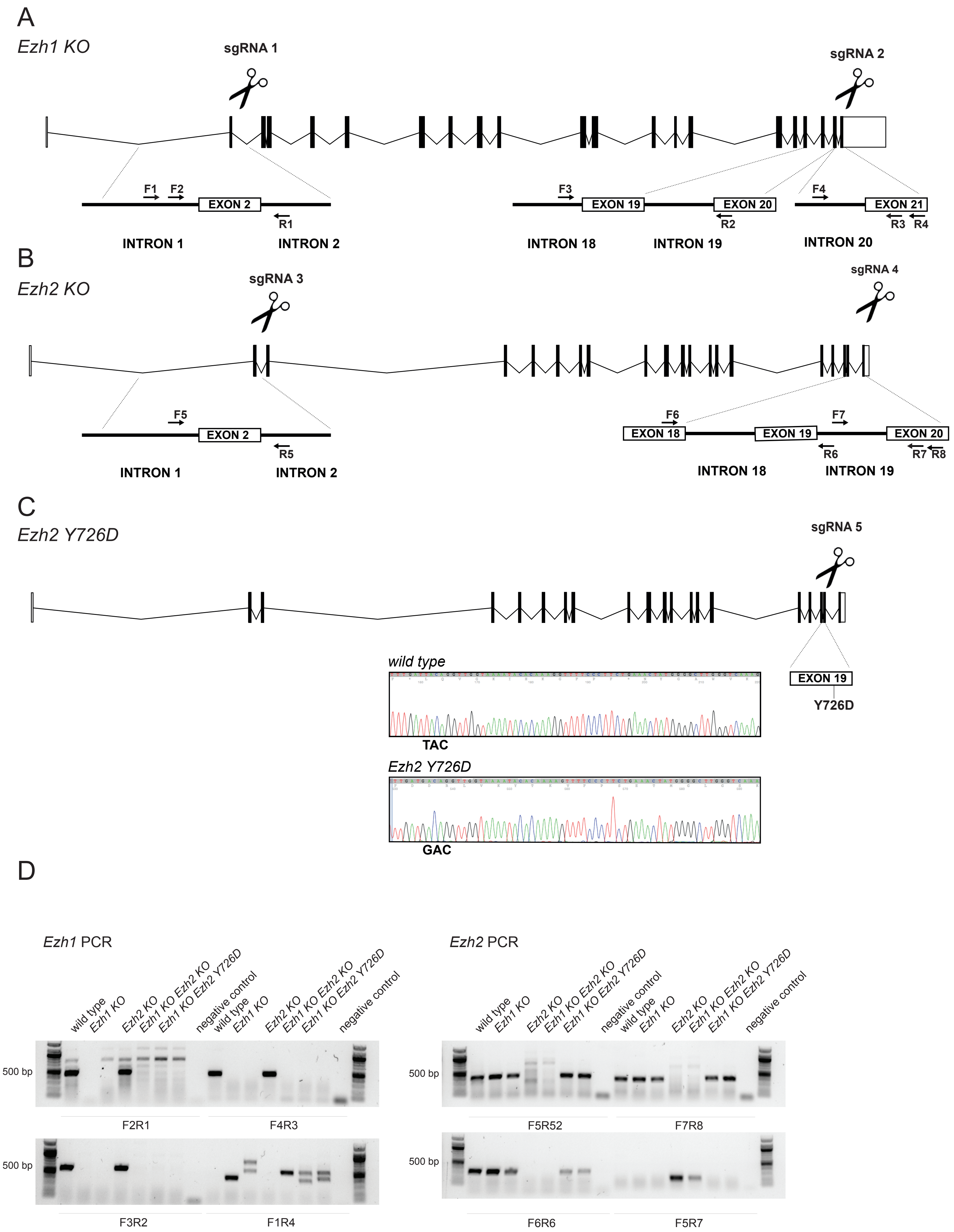
A
Ezh1 KO
sgRNA 1
sgRNA 2
F1 F2
EXON 2
R1
INTRON 1
INTRON 2
F3
EXON 19
EXON 20
R2
F4
EXON 21
R3 R4
INTRON 18
INTRON 19
INTRON 20
B
Ezh2 KO
sgRNA 3
sgRNA 4
F5
EXON 2
R5
INTRON 1
INTRON 2
F6
EXON 18
EXON 19
F7
EXON 20
R6
R7 R8
INTRON 18
INTRON 19
C
Ezh2 Y726D
sgRNA 5
wild type
TAC
Ezh2 Y726D
GAC
EXON 19
Y726D
D
Ezh1 PCR
wild type
Ezh1 KO
Ezh2 KO
Ezh1 KO Ezh2 KO
Ezh1 KO Ezh2 Y726D
negative control
500 bp
F2R1
F4R3
F3R2
F1R4
Ezh2 PCR
F5R52
F7R8
F6R6
F5R7

Supplementary Figure 1. CRISPR/Cas9 Strategies Used to Generate ESC Engineered Mouse Lines. Related to Figure 1.

(A–C) Schematic representation of CRISPR/Cas9 strategies used to generate *Ezh1* KO (A), *Ezh2* KO (B), and *Ezh2* Y726D knock-in (C) mutant mESCs. The position of sgRNAs used for the targeting is highlighted with scissors. PCR primers used for screening individual clones are indicated with arrows. Sanger sequencing results (C; bottom panels) are presented as validation for the introduction of the desired point mutation (726 TAC > GAC) in *Ezh2* Y726D cells.

(D) Agarose gel electrophoresis showing the results of PCR reactions for the *Ezh1* and *Ezh2* genes using the different primers combinations highlighted in (A) and (B), respectively, showing the genotype of the indicated cell lines.

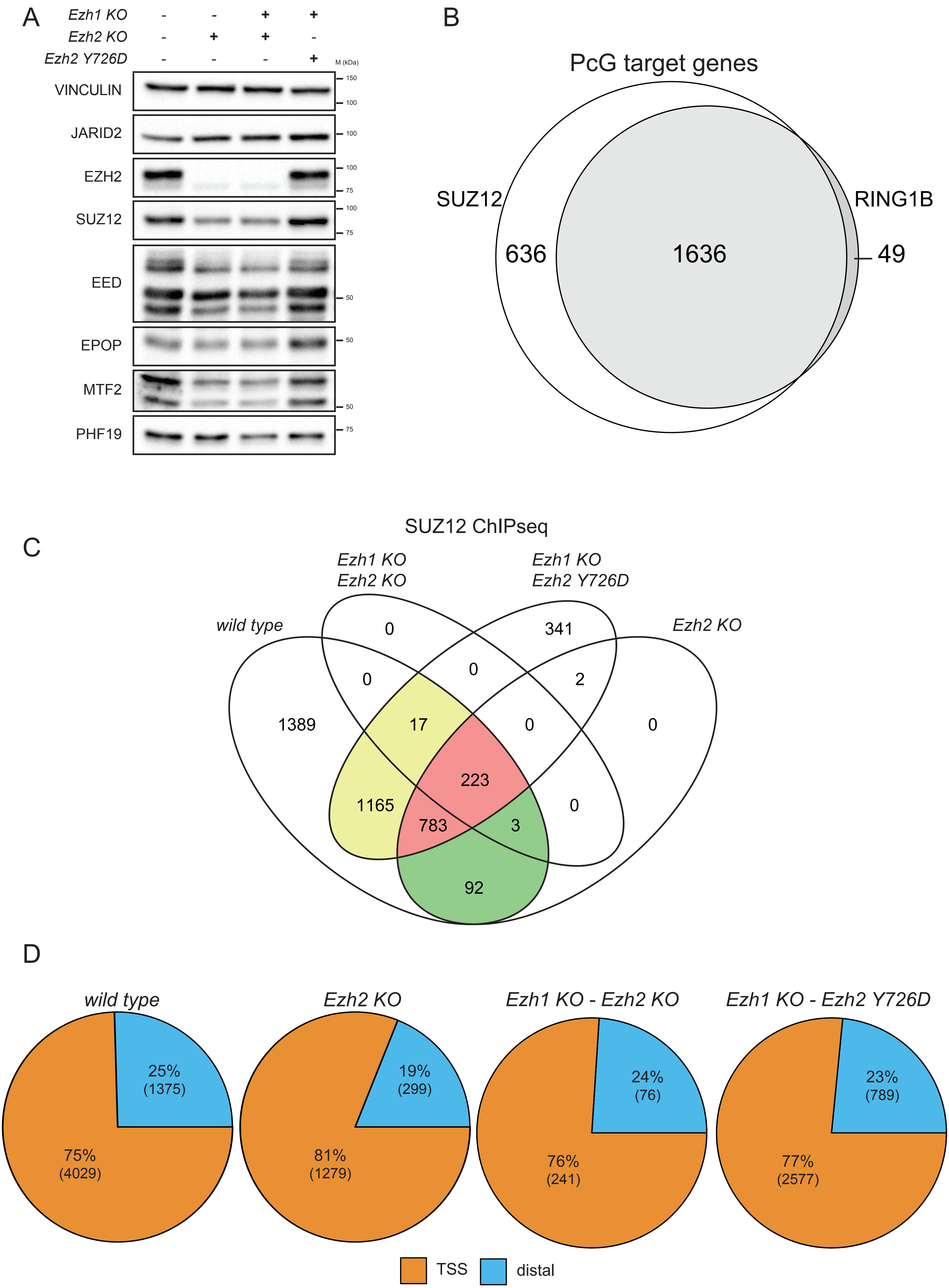
A
Ezh1 KO - - + +
Ezh2 KO - + + -
Ezh2 Y726D - - - +
M (kDa)
VINCULIN
JARID2
EZH2
SUZ12
EED
EPOP
MTF2
PHF19
150
100
75
50
B
PcG target genes
SUZ12
RING1B
636
1636
49
C
SUZ12 ChIPseq
Ezh1 KO
Ezh2 KO
Ezh1 KO
Ezh2 Y726D
wild type
Ezh2 KO
0
341
0
0
2
1389
17
0
0
223
1165
0
783
3
92
D
wild type
Ezh2 KO
Ezh1 KO - Ezh2 KO
Ezh1 KO - Ezh2 Y726D
25%
(1375)
75%
(4029)
19%
(299)
81%
(1279)
24%
(76)
76%
(241)
23%
(789)
77%
(2577)
TSS
distal

Supplementary Figure 2. Genome-wide Overlap and Distribution of SUZ12 Peaks at Promoter Regions. Related to Figure 1.

(A) Western blot analysis of PRC2 subunits using the indicated antibodies with protein extracts obtained from WT, *Ezh2* KO, *Ezh1/2* dKO and *Ezh1* KO *Ezh2* Y726D mouse ESC lines. Vinculin was used as loading control.

(B) Venn diagrams representing the overlap of SUZ12 and RING1B target genes indentified by ChIP-seq analysis in WT mESCs.

(C) Venn diagrams representing the overlap of SUZ12 peaks at promoter regions identified by ChIP-seq analysis performed in the indicated cell lines.

(D) Pie charts showing the distribution in percentage and number of SUZ12 peaks identified by ChIP-seq analysis relative to TSS regions (± 5 kb) and distal genomic regions (distal).

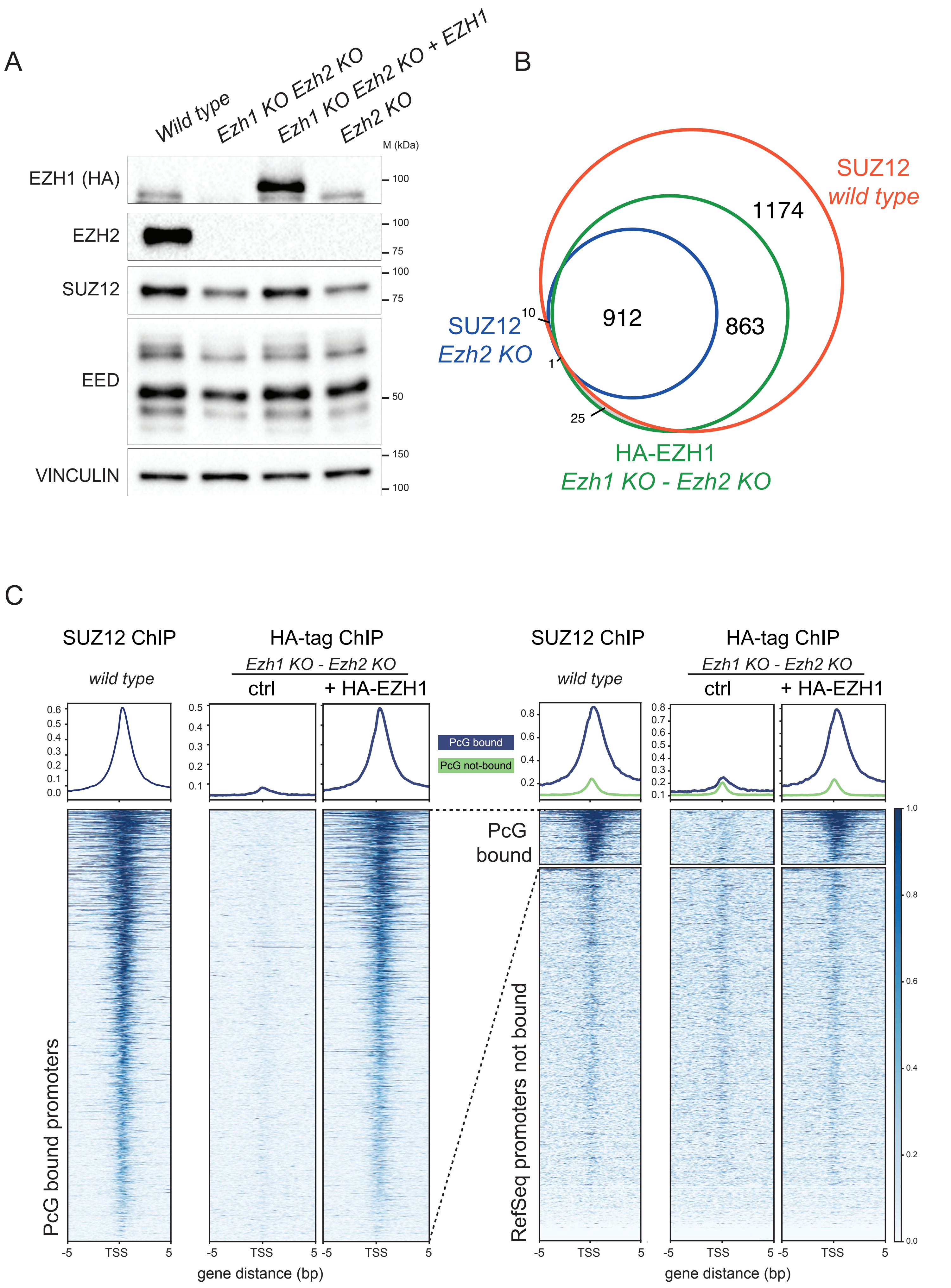
A
Wild type
Ezh1 KO Ezh2 KO
Ezh1 KO Ezh2 KO + EZH1
Ezh2 KO
M (kDa)
EZH1 (HA)
100
EZH2
100
75
SUZ12
100
75
EED
50
VINCULIN
150
100
B
SUZ12
wild type
1174
912
863
10
1
25
SUZ12
Ezh2 KO
HA-EZH1
Ezh1 KO - Ezh2 KO
C
SUZ12 ChIP
wild type
HA-tag ChIP
Ezh1 KO - Ezh2 KO
ctrl
+ HA-EZH1
PcG bound
PcG not-bound
PcG bound
PcG bound promoters
RefSeq promoters not bound
-5
TSS
5
gene distance (bp)

Supplementary Figure 3. Genome-wide Distribution of EZH1 Chromatin Association. Related to Figure 1.

(A) Western blot analysis using the indicated antibodies with protein extracts obtained from WT, *Ezh2* KO, *Ezh1/2* dKO and *Ezh1/2* dKO rescued with hEZH1 mouse ESC lines. Vinculin was used as loading control.

(B) Venn diagrams representing the overlap between SUZ12 peaks in wild type and *Ezh2* KO cells and HA-tag peaks in *Ezh1*KO *Ezh2* KO rescued with HA-tagged EZH1 at promoter regions identified by ChIP-seq analysis.

(C) Heatmaps representing the normalized SUZ12 and HA-tag ChIP-seq intensities ± 5 kb around TSS of Polycomb-bound promoters (left panels) and of all annotated TSS in RefSeq (right panels) in the indicated cell lines. Anti-HA ChIP in untransfected *Ezh1*KO *Ezh2* KO cells was used as negative control for HA-tag ChIP. Promoters were ranked according to their intensities in WT mESCs. Enrichment plots representing the average distribution of SUZ12 and HA-tag ± 5 kb around TSS are shown in the upper panels.

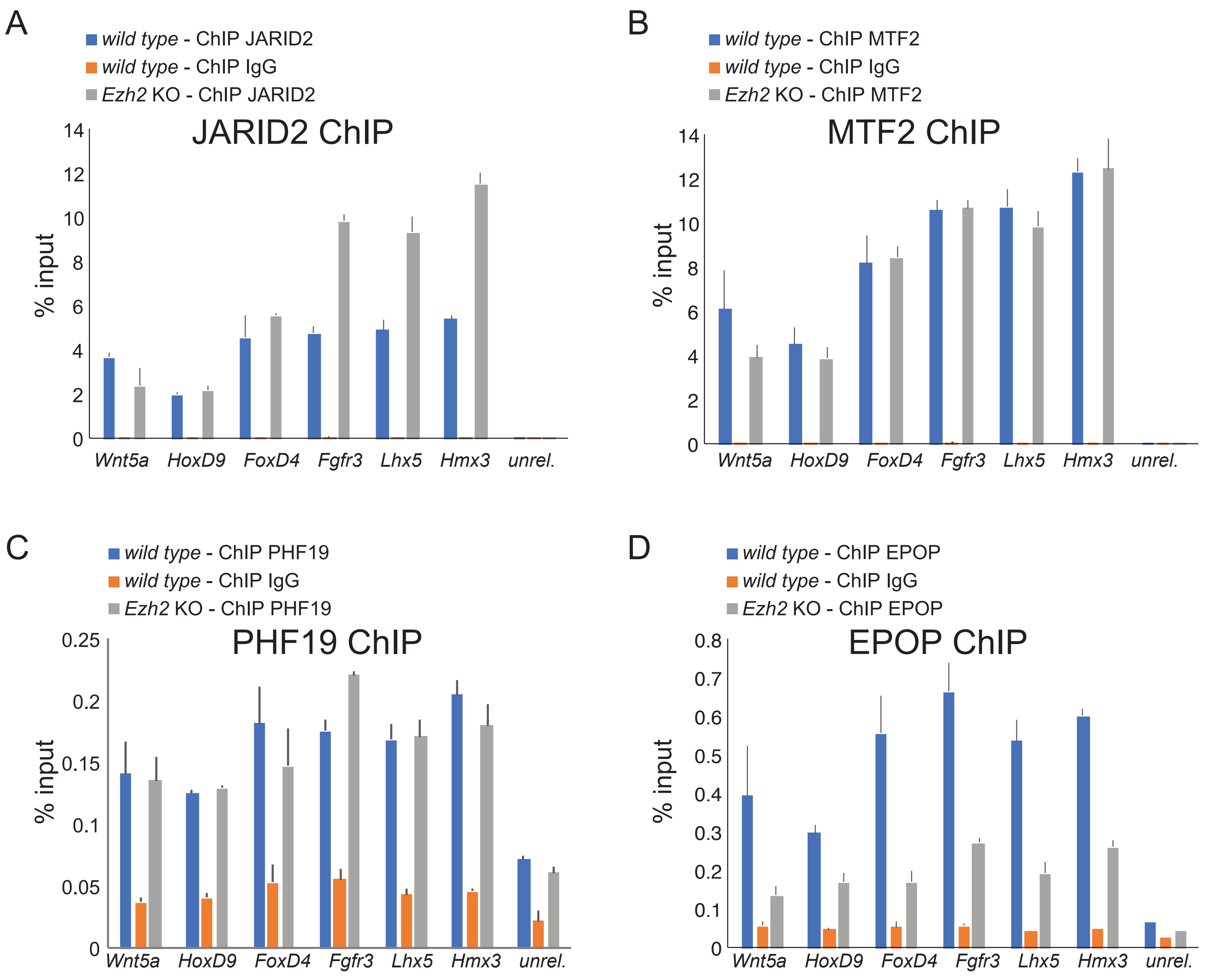

Supplementary Figure 4. Locus-specific Distribution of PRC2 ancillary subunits at promoter regions. Related to Figure 1.

ChIP-qPCR analyses of JARID2 (panel A), MTF2 (panel B), PHF19 (panel C) and EPOP (panel D) in Wild type and *Ezh2* KO cells. IgG rabbit served as negative control. Enrichments are normalized to % INPUT. Data are represented as mean ± SEM.

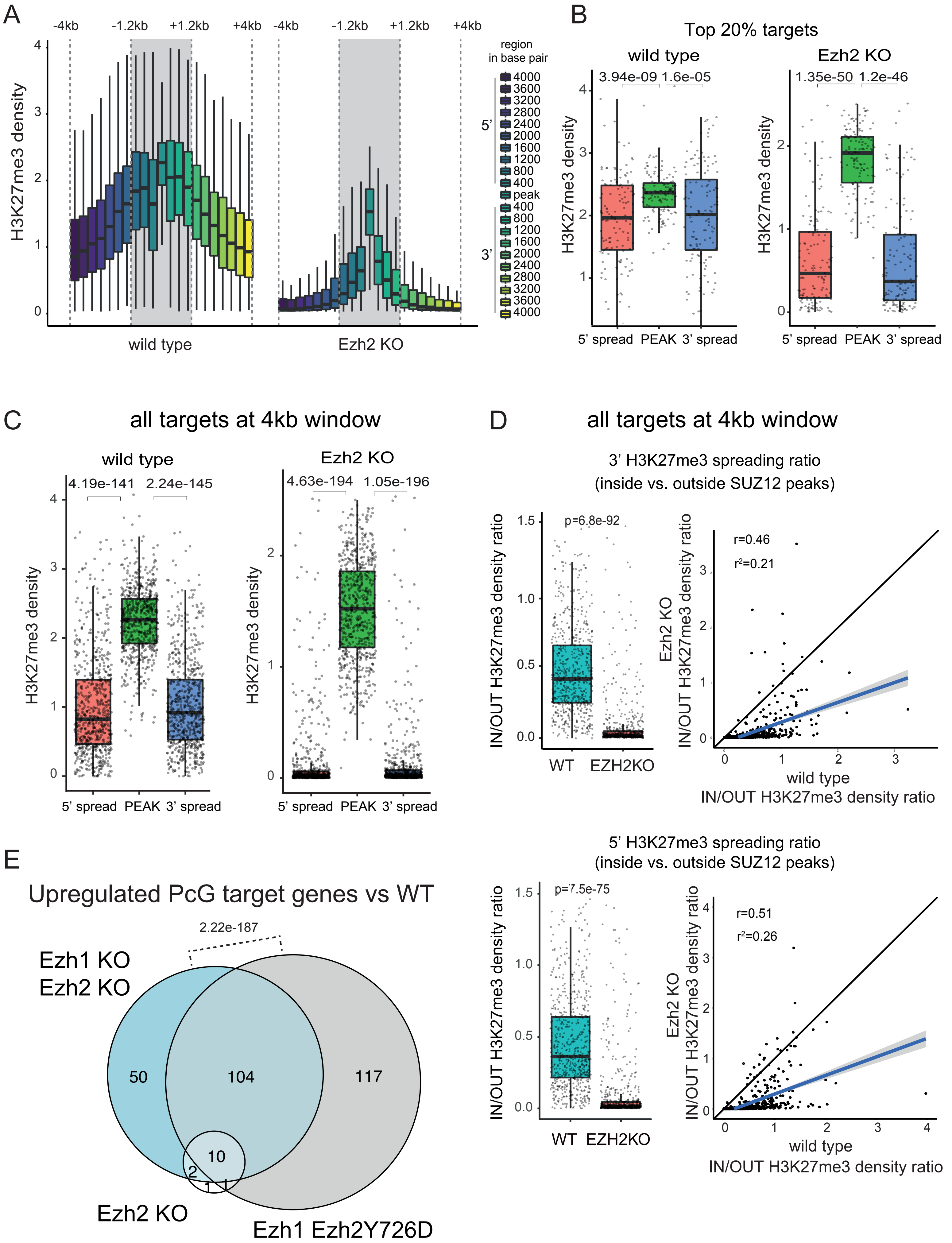
A
-4kb
-1.2kb
+1.2kb
+4kb
H3K27me3 density
region in base pair
4000
3600
3200
2800
2400
2000
1600
1200
800
400
peak
5'
3'
wild type
Ezh2 KO
B
Top 20% targets
wild type
Ezh2 KO
3.94e-09
1.6e-05
1.35e-50
1.2e-46
5' spread
PEAK
3' spread
C
all targets at 4kb window
4.19e-141
2.24e-145
4.63e-194
1.05e-196
D
all targets at 4kb window
3' H3K27me3 spreading ratio
(inside vs. outside SUZ12 peaks)
IN/OUT H3K27me3 density ratio
p=6.8e-92
WT
EZH2KO
r=0.46
r²=0.21
Ezh2 KO IN/OUT H3K27me3 density ratio
wild type
IN/OUT H3K27me3 density ratio
5' H3K27me3 spreading ratio
(inside vs. outside SUZ12 peaks)
p=7.5e-75
r=0.51
r²=0.26
E
Upregulated PcG target genes vs WT
2.22e-187
Ezh1 KO
Ezh2 KO
50
104
117
10
2
1
1
Ezh2 KO
Ezh1 Ezh2Y726D

Supplementary Figure 5. PRC2-EZH1 Mediated H3K27me3 Deposition and Target Repression. Related to Figure 2.

(A) Boxplots representing H3K27me3 density distribution in WT and *Ezh2* KO cells within PRC2 peaks as well as considering different windows, from ± 0.4 to 4 kb, outside the 5′ and 3′ ends.

(B) Boxplots representing the distribution of the H3K27me3 density ratio between the H3K27me3 density inside PRC2 peaks and at 5′ (left panel) or 3′ (right panels) spreading regions at top 20% H3K27me3 enriched genes in *Ezh2* KO cells. P-values were determined using a Student's *t*-test.

(C) Boxplots representing the distribution of the H3K27me3 density ratio between the H3K27me3 density inside PRC2 peak and at 5′ (left panel) or 3′ (right panels) spreading regions considering a ± 4 kb window. P-values were determined using a Student's *t*-test.

(D) Left panels, boxplots representing the distribution of the H3K27me3 density ratio between the H3K27me3 density inside PRC2 peaks and at 3′ (upper panel) or 5′ (bottom panel) spreading regions considering a ± 4 kb window. P-values were determined using a Student's *t*-test. Right panels, the correlation for the IN/OUT H3K27me3 density ratio between WT and *Ezh2* KO cells. The linear correlation coefficient (r) and coefficient of determination (r^2) are shown in the graphs.

(E) Venn diagrams representing the overlap between upregulated genes in *Ezh2* KO, *Ezh1* KO *Ezh2* KO and *Ezh1* KO *Ezh2* Y726D cells compared to Wild type. P-values were determined by hypergeometric distribution.

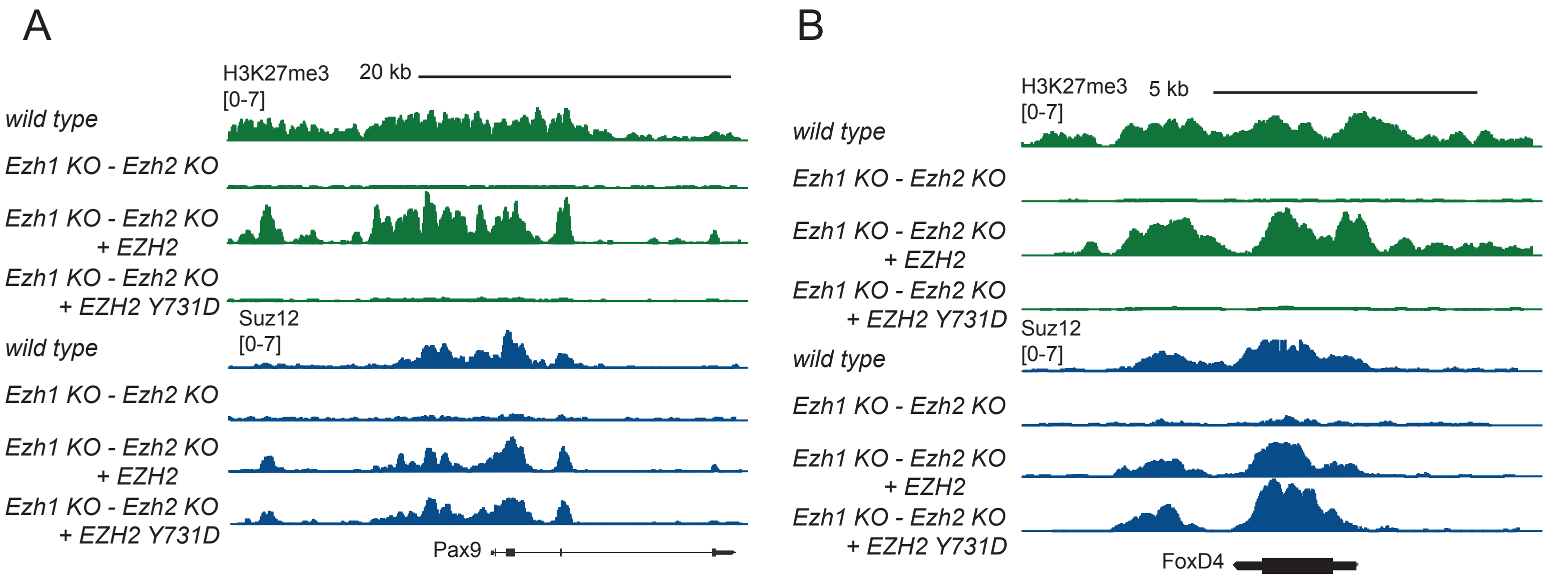

Supplementary Figure 6. SUZ12 is Recruited *De Novo* to Chromatin in the Absence of H3K27me3. Related to Figure 3.

(A, B) Representative genomic snapshots of H3K27me3 and SUZ12 ChIP-seq analyses performed in the indicated cell lines at *Pax9* (A) and *FoxD4* loci (B); these data extend those presented in Figure 3D.

A

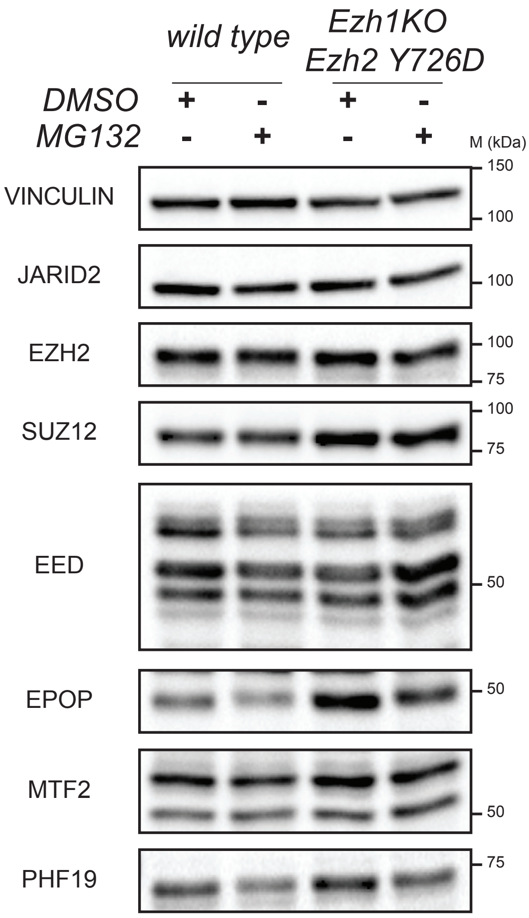
wild type
Ezh1KO Ezh2 Y726D
DMSO + - + -
MG132 - + - +
M (kDa)
VINCULIN
JARID2
EZH2
SUZ12
EED
EPOP
MTF2
PHF19
150
100
75
50

B

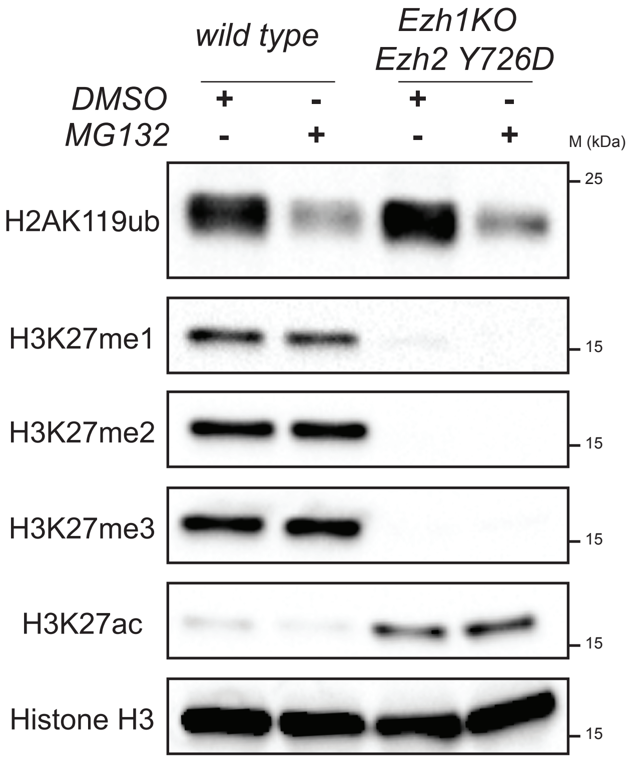
wild type
Ezh1KO Ezh2 Y726D
DMSO + - + -
MG132 - + - +
M (kDa)
H2AK119ub
H3K27me1
H3K27me2
H3K27me3
H3K27ac
Histone H3
25
15

C

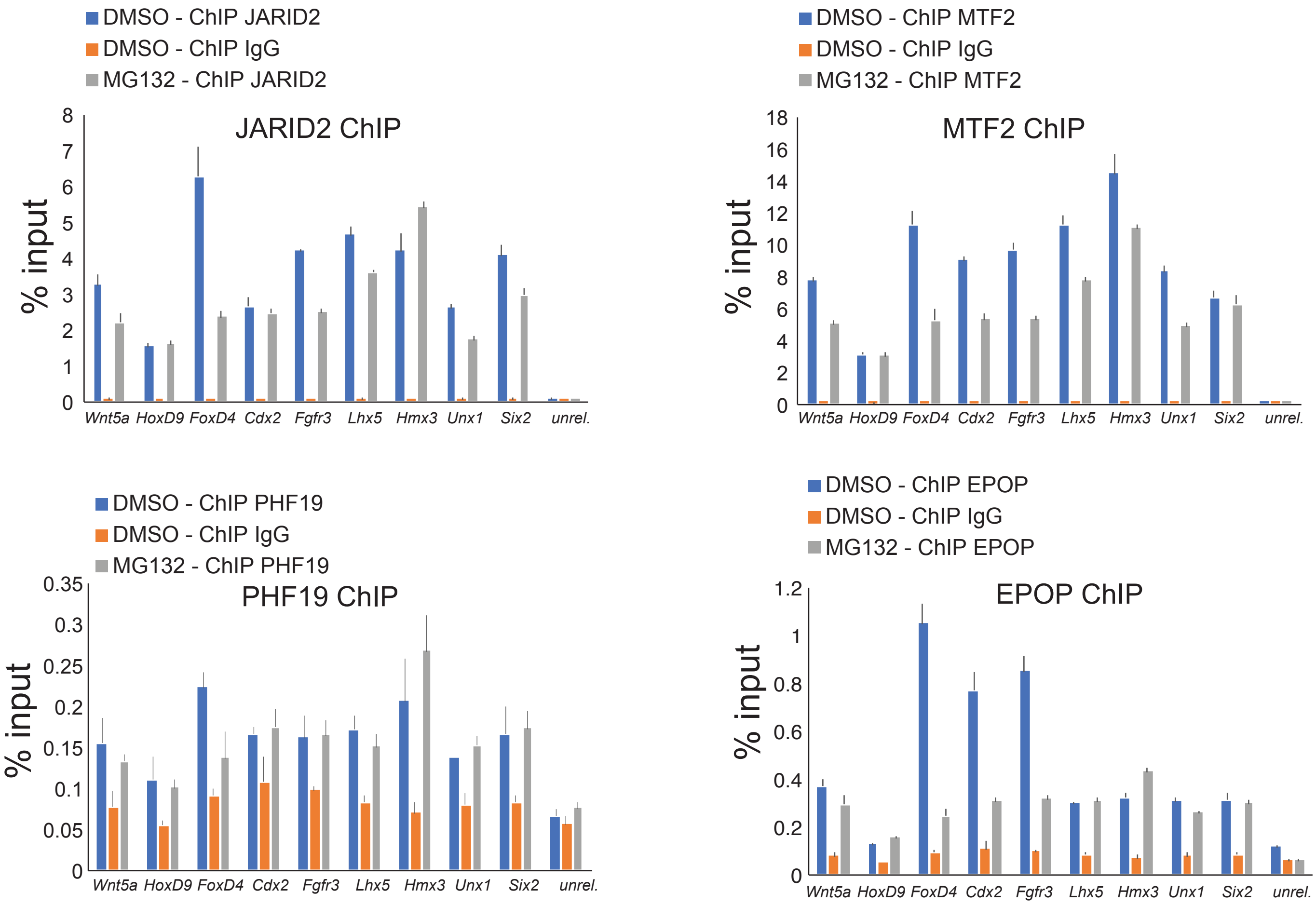
DMSO - ChIP JARID2
DMSO - ChIP IgG
MG132 - ChIP JARID2
JARID2 ChIP
% input
DMSO - ChIP MTF2
DMSO - ChIP IgG
MG132 - ChIP MTF2
MTF2 ChIP
DMSO - ChIP PHF19
DMSO - ChIP IgG
MG132 - ChIP PHF19
PHF19 ChIP
DMSO - ChIP EPOP
DMSO - ChIP IgG
MG132 - ChIP EPOP
EPOP ChIP
Wnt5a HoxD9 FoxD4 Cdx2 Fgfr3 Lhx5 Hmx3 Unx1 Six2 unrel.

Supplementary Figure 7. PRC2 Ancillary Subunits Remain Unaltered Upon MG132 Treatment. Related to Figure 4.

(A) Western blot analysis of PRC2 subunits using the indicated antibodies with protein extracts obtained from WT and *Ezh1* KO *Ezh2* Y726D mouse ESC lines upon treatment with DMSO or MG132. Vinculin was used as loading control.

(B) Western blot analysis using the indicated antibodies with protein extracts obtained from WT and *Ezh1* KO *Ezh2* Y726D mouse ESC lines upon treatment with DMSO or MG132. Histone H3 was used as loading control.

(C) ChIP-qPCR analyses of JARID2, MTF2, PHF19 and EPOP in Wild type cells upon treatment with DMSO or MG132. IgG rabbit served as negative control. Enrichments are normalized to % INPUT. Data are represented as mean ± SEM.

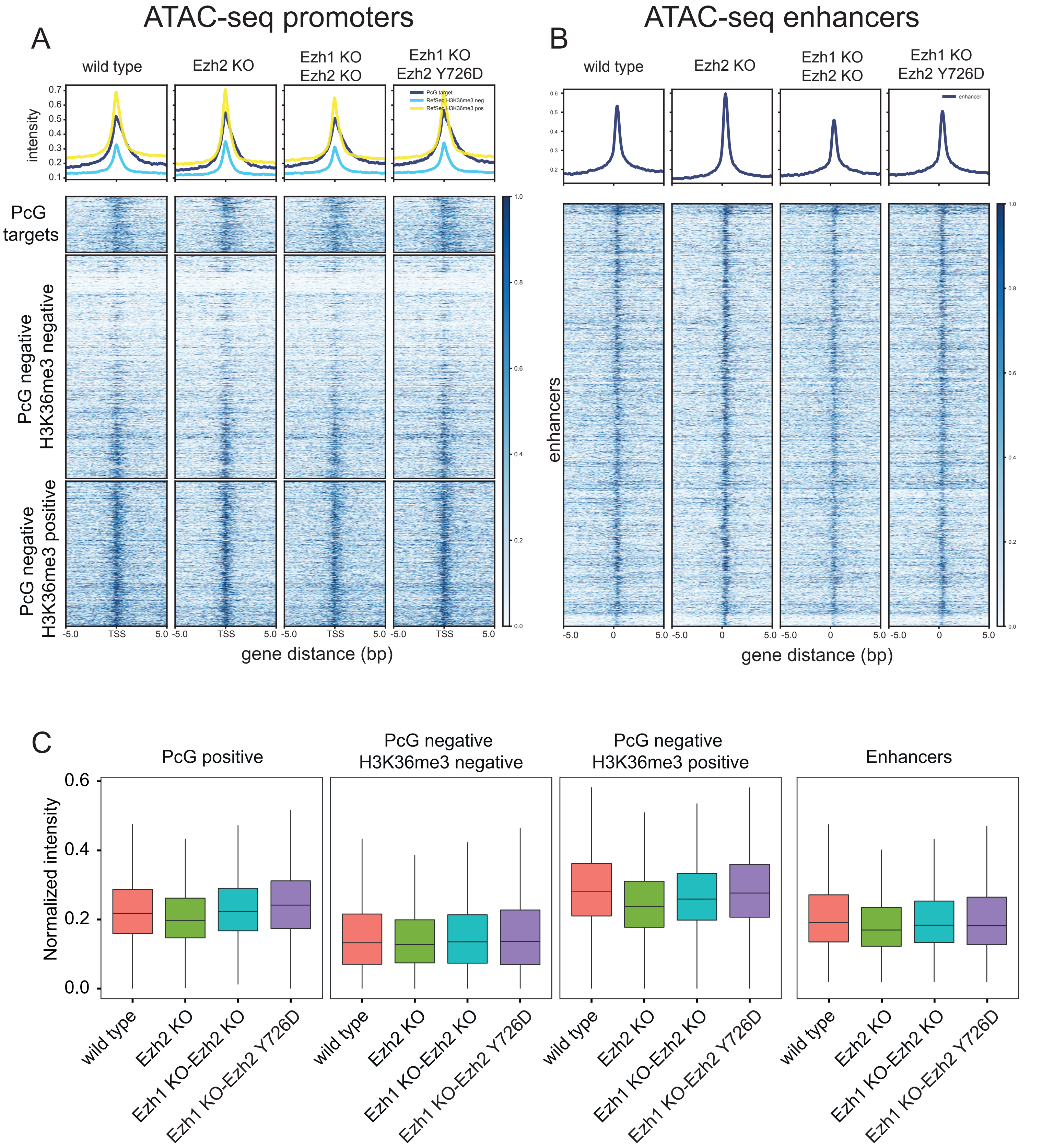

A
ATAC-seq promoters
wild type
Ezh2 KO
Ezh1 KO
Ezh2 KO
Ezh1 KO
Ezh2 Y726D
intensity
PcG target
RefSeq H3K36me3 neg
RefSeq H3K36me3 pos
PcG targets
PcG negative H3K36me3 negative
PcG negative H3K36me3 positive
-5.0
TSS
5.0
gene distance (bp)
B
ATAC-seq enhancers
wild type
Ezh2 KO
Ezh1 KO
Ezh2 KO
Ezh1 KO
Ezh2 Y726D
enhancer
enhancers
-5.0
0
5.0
gene distance (bp)
C
PcG positive
PcG negative
H3K36me3 negative
PcG negative
H3K36me3 positive
Enhancers
Normalized intensity
0.6
0.4
0.2
0.0
wild type
Ezh2 KO
Ezh1 KO-Ezh2 KO
Ezh1 KO-Ezh2 Y726D

Supplementary Figure 8. Loss of PRC2 Activity Does Not Alter Chromatin Accessibility. Related to Figure 6.

(A) Heatmaps representing ATAC-seq profiles ± 5 kb around TSS of all annotated TSS in RefSeq in the indicated cell lines. Promoters were clustered into Polycomb targets, H3K36me3 positive and negative regions. PcG targets were ranked for the intensity of H3K27me3 deposition while the rest of RefSeq genes for ascending intensity of H3K36me3. Upper boxplots represent the ATAC-seq normalized intensities in the indicated cell lines in the different gene clusters.

(B) Heatmaps representing ATAC-seq profiles ± 5 kb around the centre of annotated ESC enhancers (see methods section) in the indicated cell lines. Upper boxplots represent the ATAC-seq normalized intensities in the indicated cell lines.

(C) Boxplots representing ATAC-seq normalized intensities in the indicated cell lines at the different promoter clusters (panel A) and at enhancers (panel B).

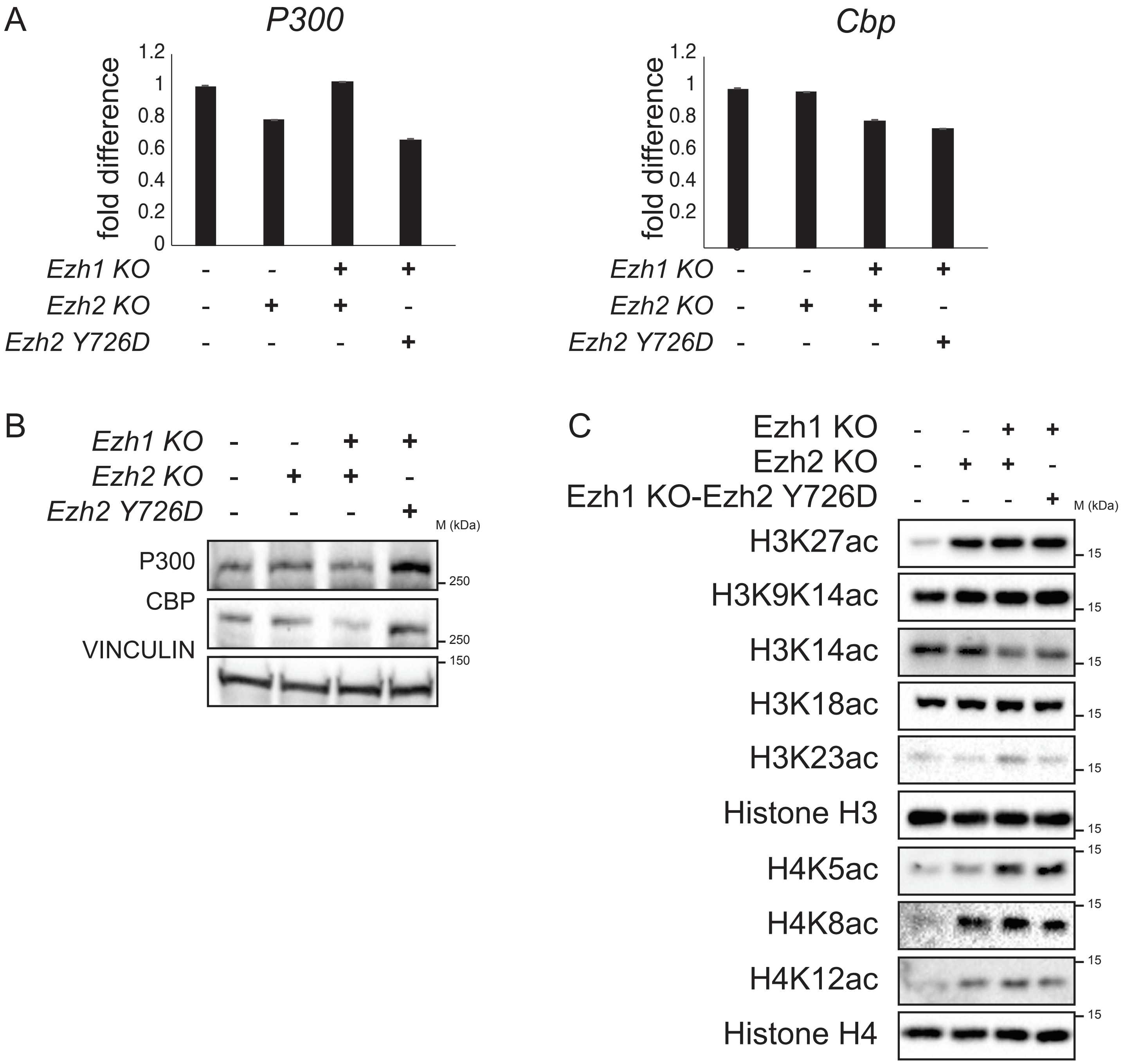

Supplementary Figure 9. Loss of PRC2 Activity Does Not Impact on P300/CBP Expression but Induces H4 N-terminal Acetylation. Related to Figure 6.

(A) Relative expression of P300 and CBP determined by Real Time PCR in the indicated cell lines. Indicated genes expression was normalized to *Rplp0* levels. Data are represented as mean ± SEM.

(B) Western blot analysis of P300 and CBP with protein extracts obtained from WT, *Ezh2* KO, *Ezh1* KO *Ezh2* KO, *Ezh1* KO *Ezh2* Y726D and *Ezh1* KO *Ezh2* R685C mouse ESC. Vinculin was used as loading control.

(C) Western blot analysis of different H3/H4 acetylated lysine residues with protein extracts obtained from WT, *Ezh2* KO, *Ezh1* KO *Ezh2* KO and *Ezh1* KO *Ezh2* Y726D mouse ESC. Histones H3 and H4 were used as loading controls.

A

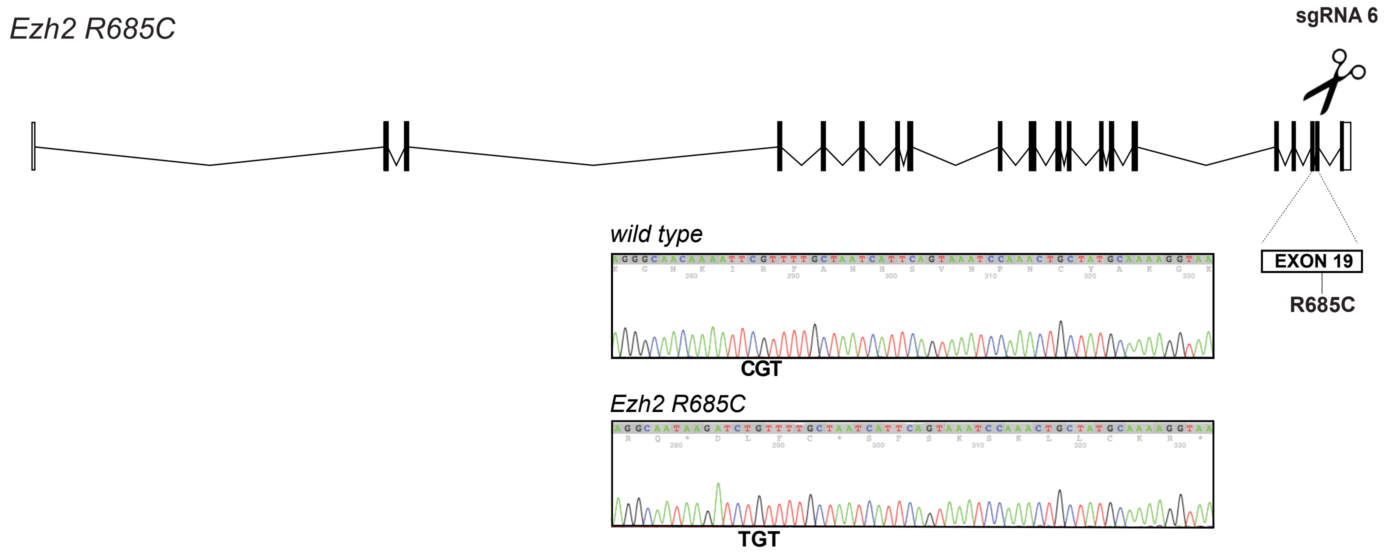

B

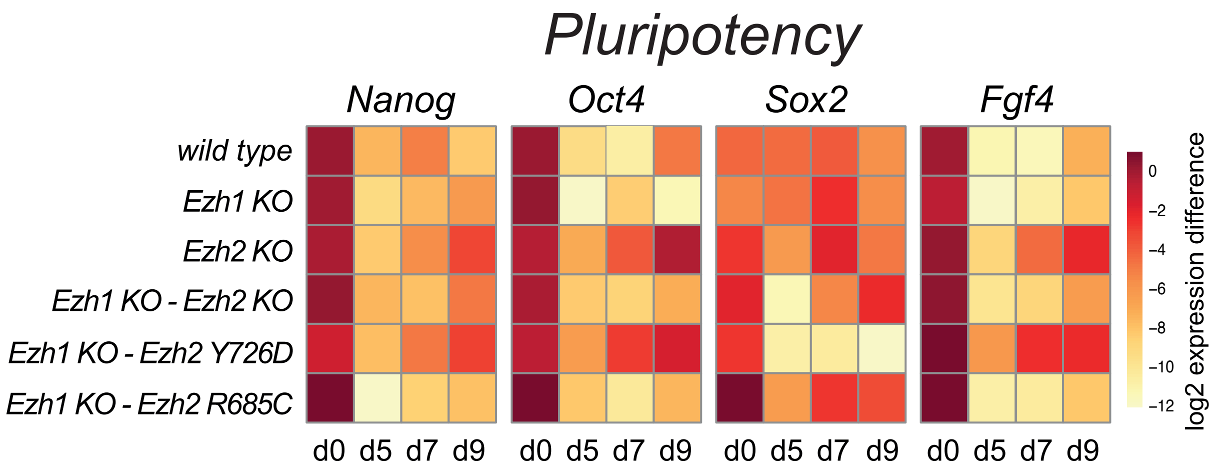

Supplementary Figure 10. Downregulation of Pluripotency Markers is Not Affected in PRC2 Mutants. Related to Figure 7.

(A) Schematic representation of CRISPR/Cas9 strategy used to generate *Ezh2* R685C knock-in mutant mESCs. The position of sgRNA used for the targeting is highlighted with scissors. Sanger sequencing results are presented as validation for the introduction of the desired point mutation (685 CGT > TGT) in *Ezh2* R685C cells.

(B) Heatmaps of qPCR relative expression analyses for the indicated pluripotency genes at d0, d5, d7 and d9 during ESC differentiation of indicated mESC lines. *Gapdh* expression served as a normalization control.

Supplementary Table 1 Lavarone *et al*.

CRISPR reagents		
target gene	name	sequence
Ezh1	sgRNA 1 F	CACCGCCATTCGTCTGCAGAACAG
	sgRNA 1 R	AAACCTGTTCTGCAGACGAATGGC
Ezh1	sgRNA 2 F	ACCGCAAGTATGTGGGCATCGAGA
	sgRNA 2 R	AAACTCTCGATGCCCACATACTTGC
Ezh2	sgRNA 3 F	CACCGATTGCTTTTAGAATAATCAT
	sgRNA 3R	AAACATGATTATTCTAAAAGCAATC
Ezh2	sgRNA 4 F	CACCGCTGATGCCCTGAAGTATGT
	sgRNA 4R	AAACACATACTTCAGGGCATCAGC
Ezh2 Y726D	sgRNA 5 F	CACCGCAGGTTGGTAAAATACACAA
	sgRNA 5 R	AAACTTGTGTATTTTACCAACCTGC
Ezh2 Y726D	Y726D ssODN	TAGTGACTGGTCAGTTAAAAGAATGCACCCTCCAATGATGGCAGACCTGTCAAAAAACTTACAAACAGCCTTGGATCCAAGCCCCATAGTTTCAGAAGGGAAAACTTTTGTGTATTTTACCAACCTGTCATCAAAAAACAACTCTTCACCAGTCTGGATAGCCCTCTTAGCAAAGATGCCTATCCTGTGGTCACCATTAA
Ezh2 R685C	sgRNA 6 F	CACCGTGTGGTGGATGCAACCCGAA
Ezh2 R685C	sgRNA 6 R	AAACTTCGGGTTGCATCCACCACAC
Ezh2 R685C	R685C ssODN	CTGGAAAAATGGAACTGTTGCATCAAACTGTCAATTCCAACTTAAAAGCTTACCTTTTGCATAGCAGTTTGGATTTACTGAATGATTAGCAAAACAGATCTTATTGCCTTTTCGGGTTGCATCCACCACAAAATCTAAAGTGAAAAACATAGATAATCCAGTGACTTATTTTCAGTCATAGACCAAGGTTATTATGGCTT

PCR primers		
target gene	name	sequence
Ezh1	F1	CCCTAGGAGGAGTTTTGAAAGC
Ezh1	F2	GCATCTCTGAGGCTTGACTTC
Ezh1	F3	TAGTTTCCTGGGACACTCATG
Ezh1	F4	GCAGGAGTGACTGTTGGAATTG
Ezh1	R1	GTTCGAATACCCTGCAACTGG
Ezh1	R2	CAAAGAAGAGCTCTTCGCCA
Ezh1	R3	CGCAGCTTCTTACATCTGAACTCT
Ezh1	R4	CAGTGACATGGAACTCTTGATCGA
Ezh2	F5	CAGAGTACATGGGCTTTTCTC
Ezh2	F6	GTGGTGGATGCAACCCGAAAG
Ezh2	F7	GTAGGTTACCAGTGACAGATGCATG
Ezh2	R5	CAGCACAGCACCAAACAGTCATG
Ezh2	R6	CAGAAGGGAAAACCTTTGTG
Ezh2	R7	GCCCACAGTACTCAAGGTTC
Ezh2	R8	CACTTTGCAGCTGGTGAGAA

Supplementary Table 1. gRNAs and primers information.

List of the sequences used for designing CRISPR/Cas9 gRNAs and for PCR primers.

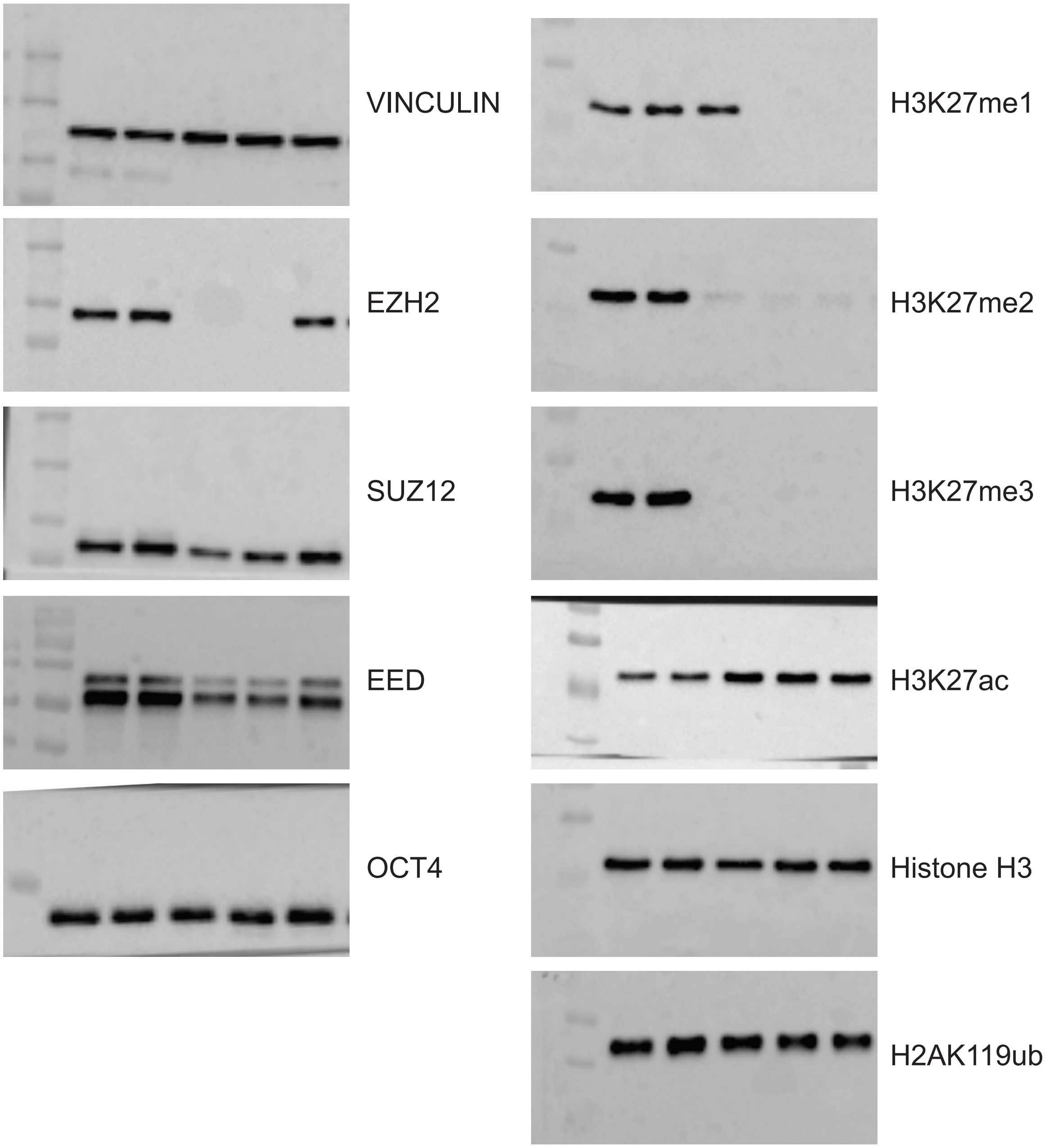

Raw data for Figure 1B

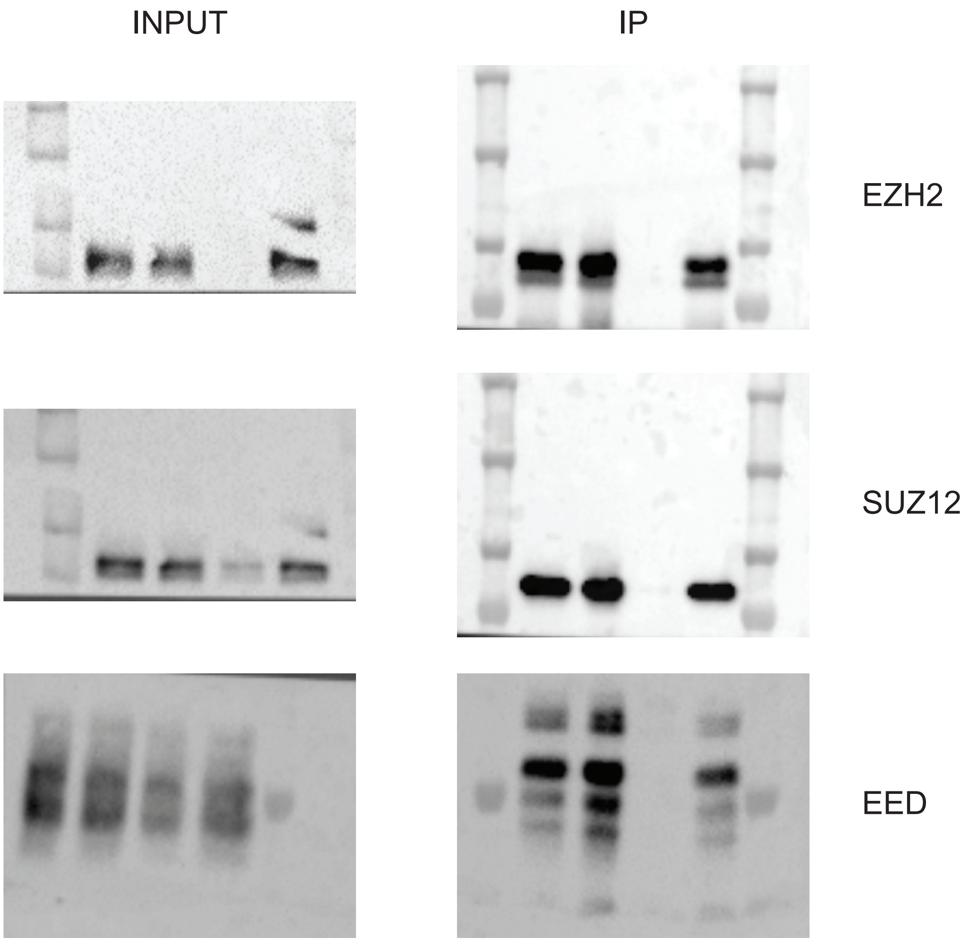

Raw data for Figure 1C

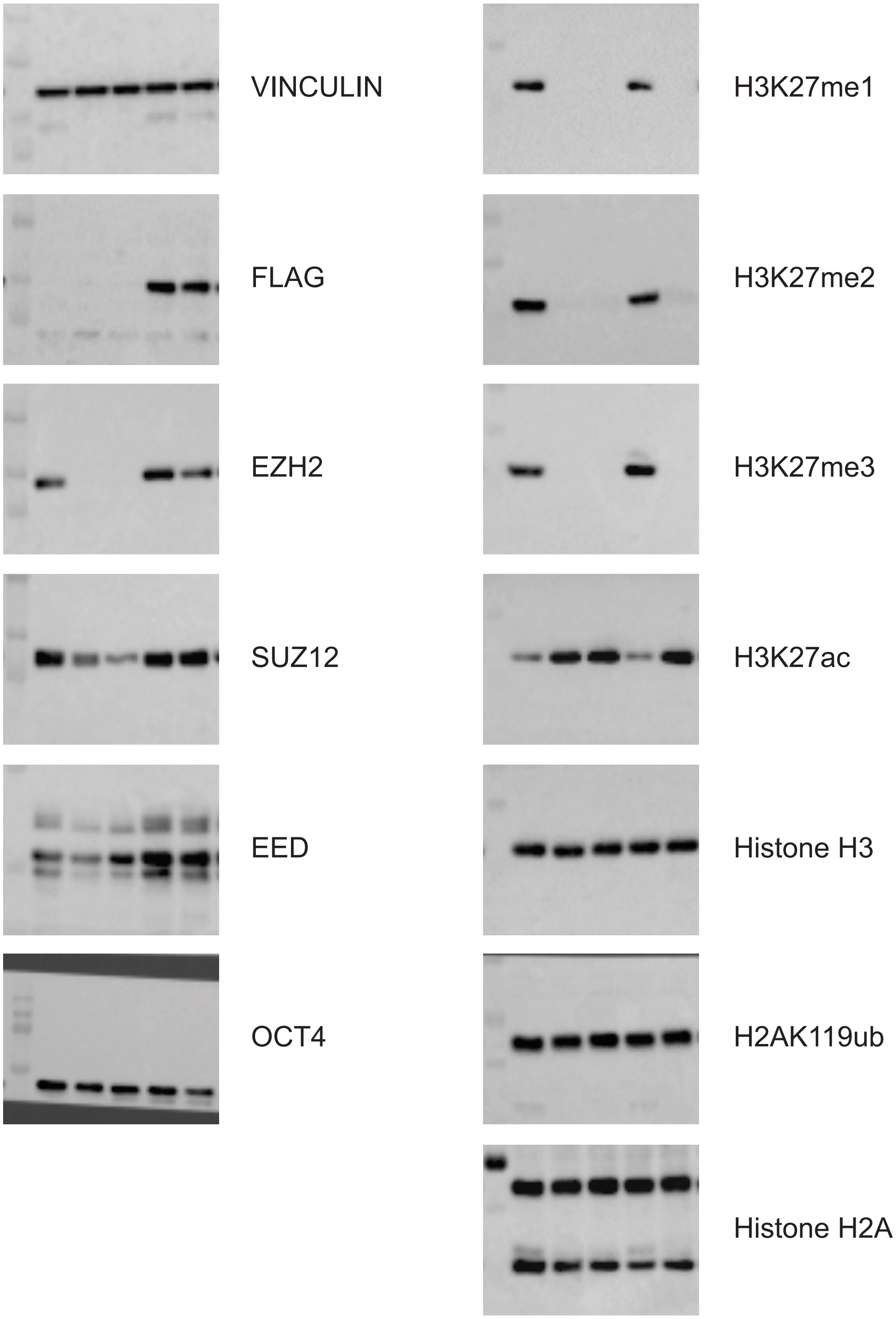

Raw data for Figure 3B

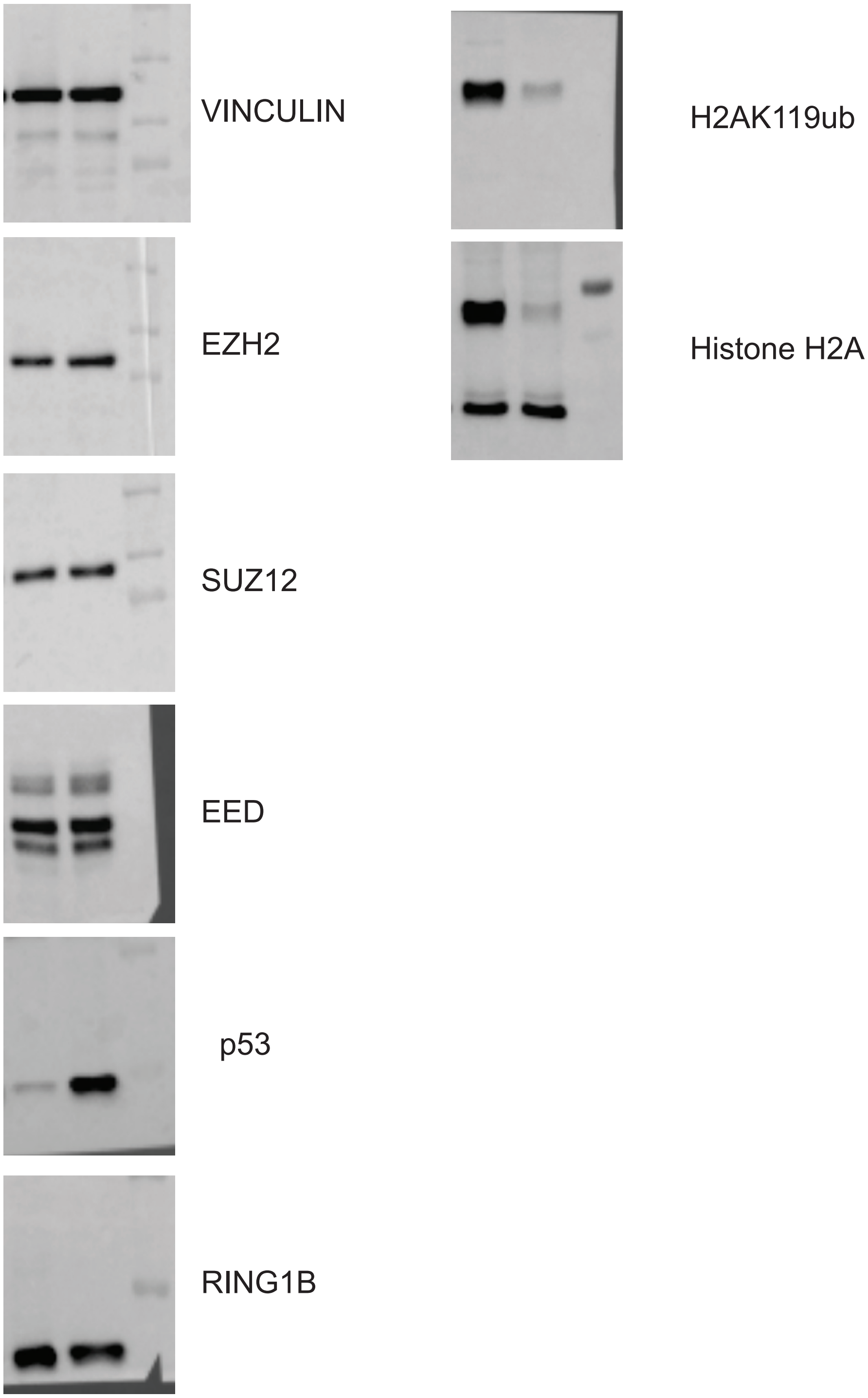

Raw data for Figure 4A

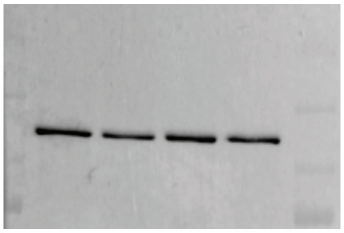

VINCULIN

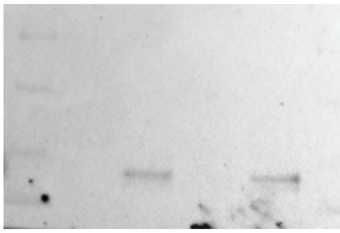

EZH2

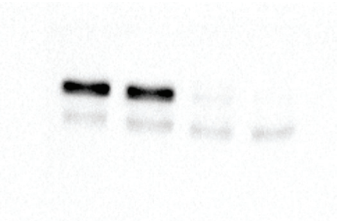

RING1B

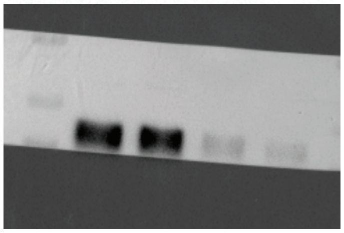

H2AK119ub

Raw data for Figure 4E

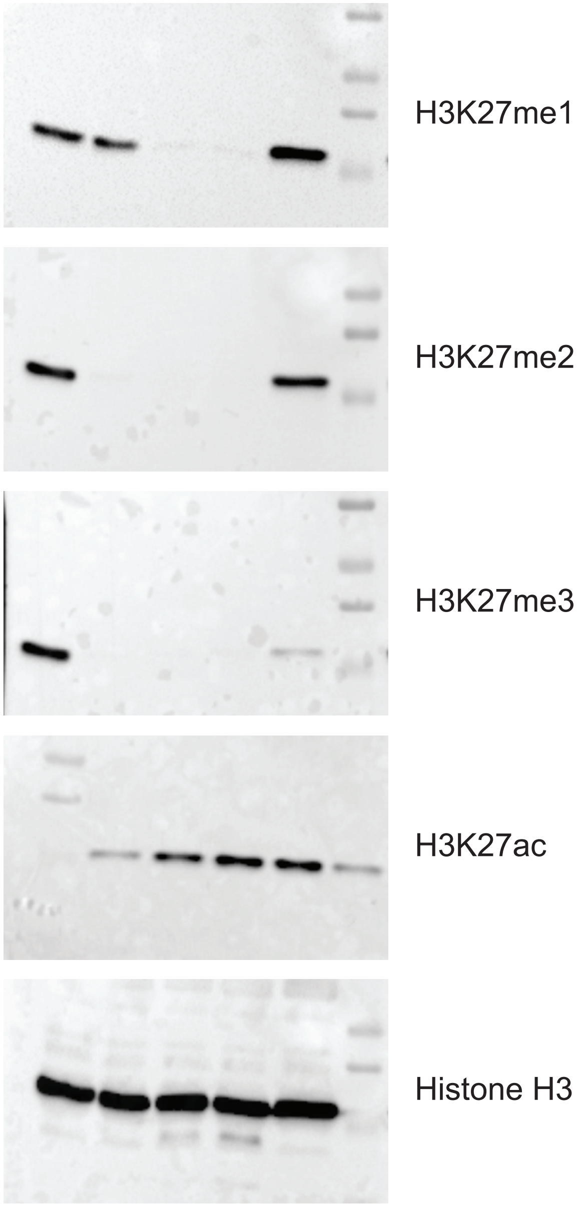

Raw data for Figure 7B

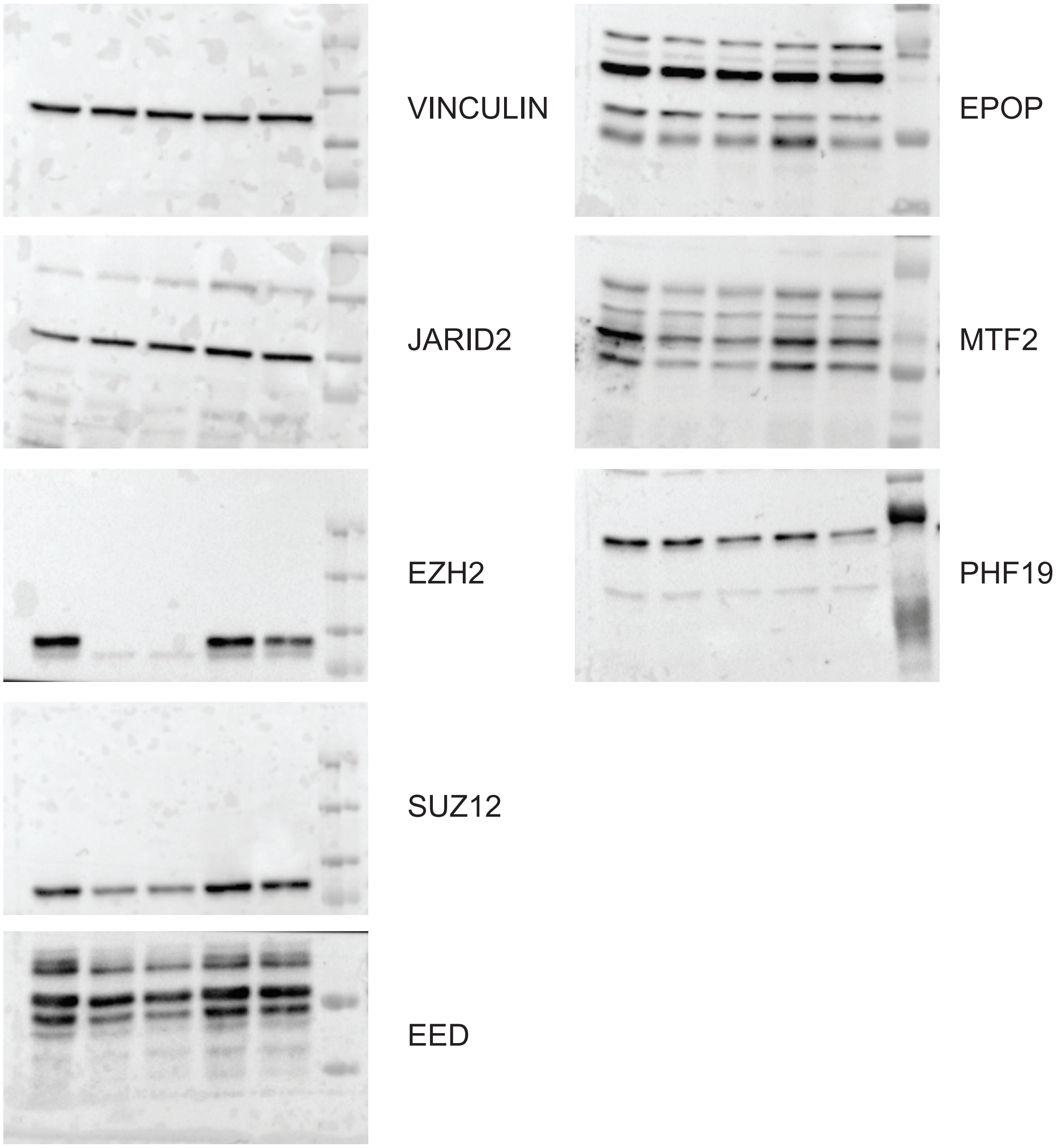

Raw data for Supplementary Figure 2A

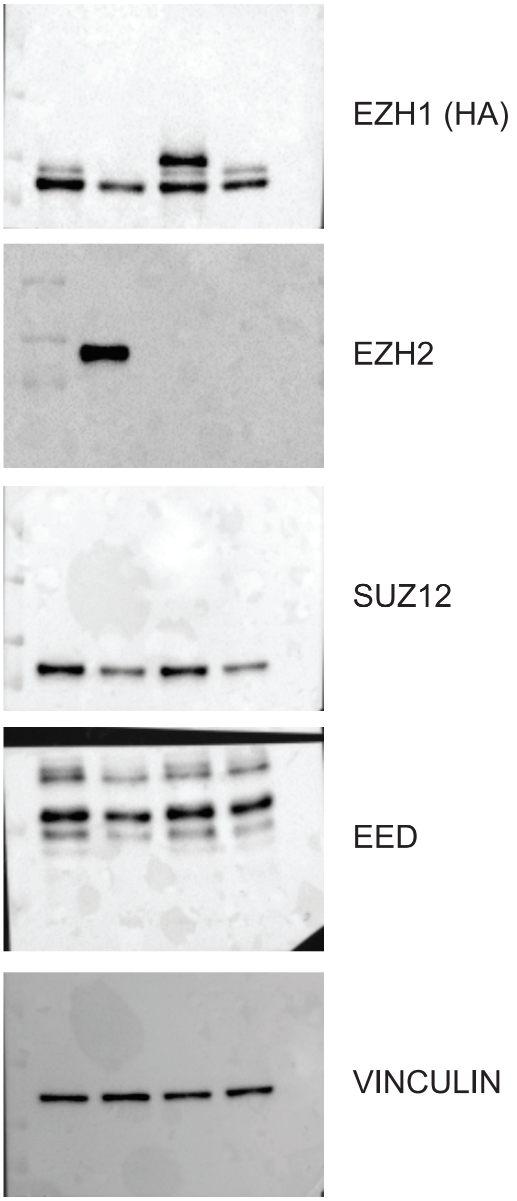

Raw data for Supplementary Figure 3A

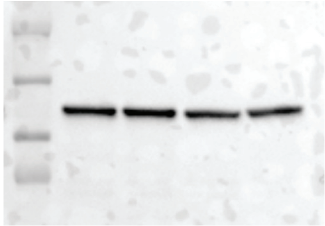

VINCULIN

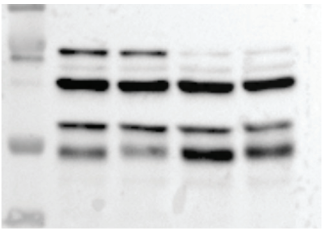

EPOP

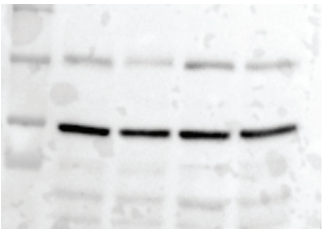

JARID2

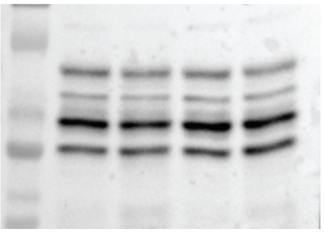

MTF2

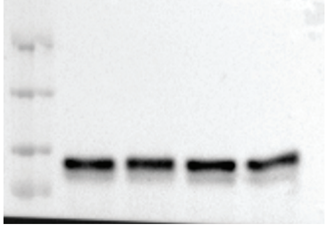

EZH2

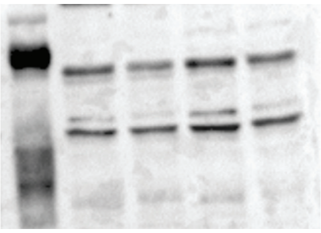

PHF19

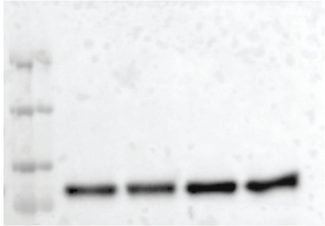

SUZ12

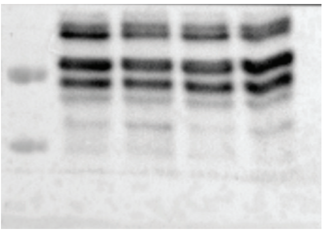

EED

Raw data for Supplementary Figure 7A

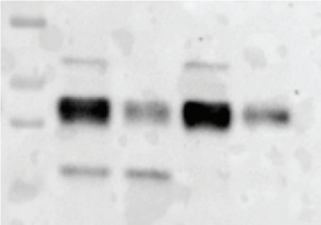

H2AK119ub

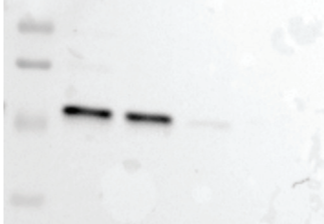

H3K27me1

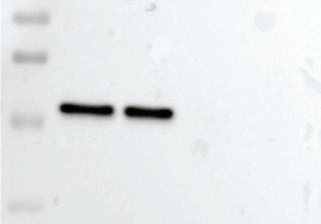

H3K27me2

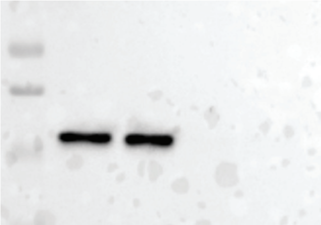

H3K27me3

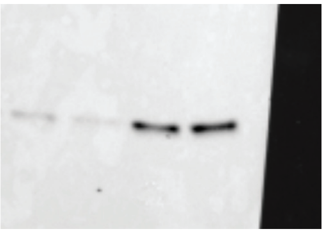

H3K27ac

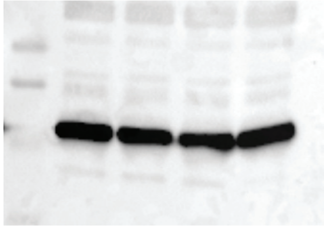

Histone H3

Raw data for Supplementary Figure 7B

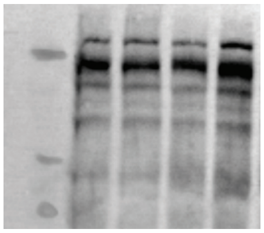

P300

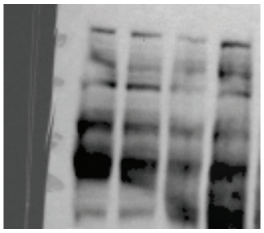

CBP

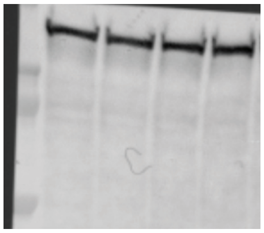

VINCULIN

Raw data for Supplementary Figure 9B

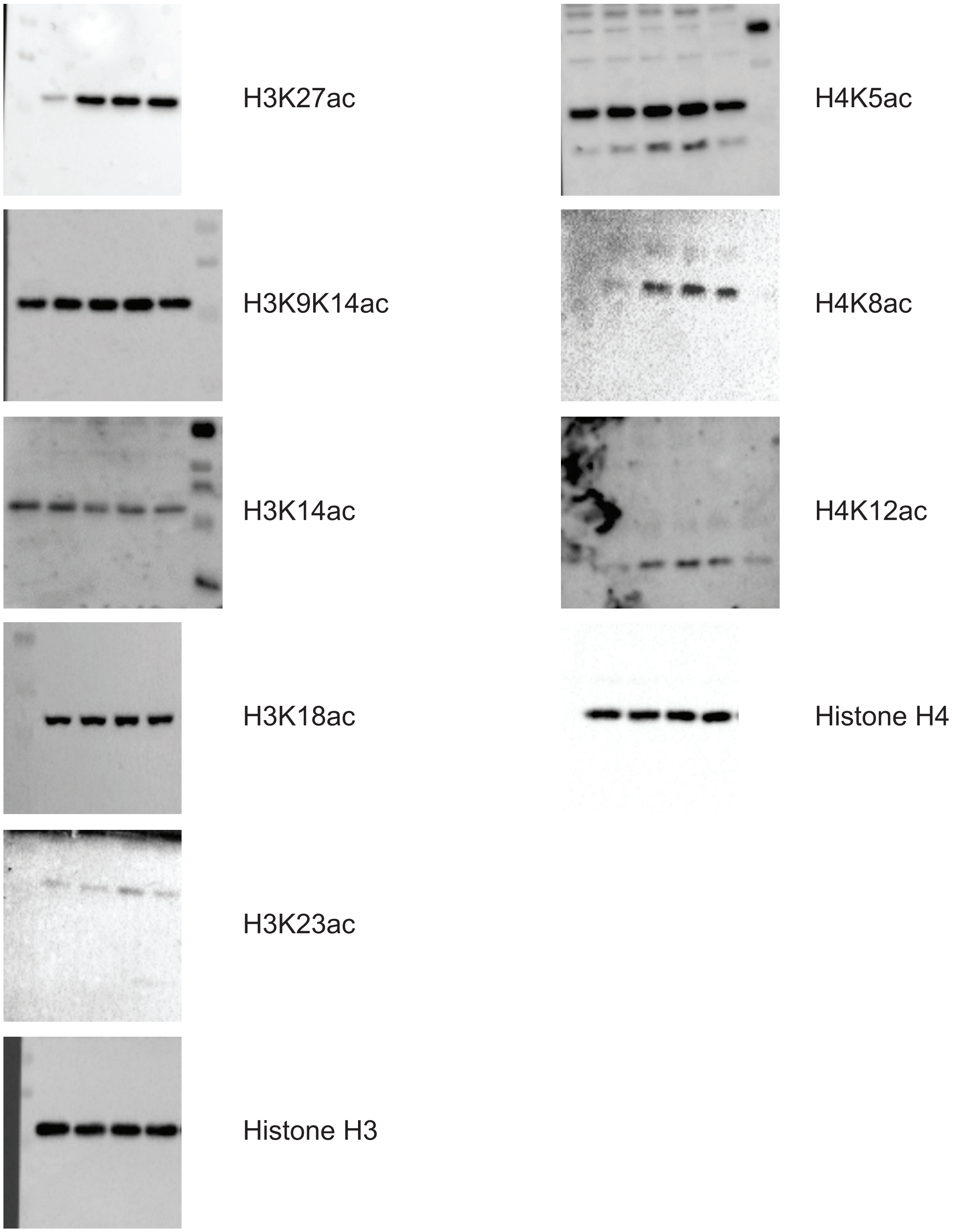

Raw data for Supplementary Figure 9C
