# Supplementary material for: Dissecting the role of H3K27 acetylation and methylation in PRC2 mediated control of cellular identity
Source: Nat Commun. 2019 Apr 11;10:1679. doi: 10.1038/s41467-019-09624-w (PMC6459869; doi:10.1038/s41467-019-09624-w)
Supplement: Supplementary file 2 — Reporting Summary [file 41467_2019_9624_MOESM2_ESM.pdf]

nature research

Corresponding author(s): Diego Pasini

Last updated by author(s): Jan 20, 2018

Reporting Summary

Nature Research wishes to improve the reproducibility of the work that we publish. This form provides structure for consistency and transparency in reporting. For further information on Nature Research policies, see Authors & Referees and the Editorial Policy Checklist.

Statistics

For all statistical analyses, confirm that the following items are present in the figure legend, table legend, main text, or Methods section.

n/a	Confirmed	
☒	☐	The exact sample size (*n*) for each experimental group/condition, given as a discrete number and unit of measurement
☐	☒	A statement on whether measurements were taken from distinct samples or whether the same sample was measured repeatedly
☐	☒	The statistical test(s) used AND whether they are one- or two-sided<br>*Only common tests should be described solely by name; describe more complex techniques in the Methods section.*
☒	☐	A description of all covariates tested
☐	☒	A description of any assumptions or corrections, such as tests of normality and adjustment for multiple comparisons
☐	☒	A full description of the statistical parameters including central tendency (e.g. means) or other basic estimates (e.g. regression coefficient) AND variation (e.g. standard deviation) or associated estimates of uncertainty (e.g. confidence intervals)
☐	☒	For null hypothesis testing, the test statistic (e.g. *F*, *t*, *r*) with confidence intervals, effect sizes, degrees of freedom and *P* value noted<br>*Give P values as exact values whenever suitable.*
☒	☐	For Bayesian analysis, information on the choice of priors and Markov chain Monte Carlo settings
☒	☐	For hierarchical and complex designs, identification of the appropriate level for tests and full reporting of outcomes
☒	☐	Estimates of effect sizes (e.g. Cohen's *d*, Pearson's *r*), indicating how they were calculated

Our web collection on statistics for biologists contains articles on many of the points above.

Software and code

Policy information about availability of computer code

Data collection	A description of the software and code has been included in the Methods.
Data analysis	A description of the software and code has been included in the Methods.

For manuscripts utilizing custom algorithms or software that are central to the research but not yet described in published literature, software must be made available to editors/reviewers. We strongly encourage code deposition in a community repository (e.g. GitHub). See the Nature Research guidelines for submitting code & software for further information.

Data

Policy information about availability of data

All manuscripts must include a data availability statement. This statement should provide the following information, where applicable:

- Accession codes, unique identifiers, or web links for publicly available datasets
- A list of figures that have associated raw data
- A description of any restrictions on data availability

All ChIP-seq datasets are available at GEO under the accession number GSE116603.

Field-specific reporting

Please select the one below that is the best fit for your research. If you are not sure, read the appropriate sections before making your selection.

☒ Life sciences ☐ Behavioural & social sciences ☐ Ecological, evolutionary & environmental sciences

Life sciences study design

All studies must disclose on these points even when the disclosure is negative.

Sample size	No sample-size calculations were performed.
Data exclusions	No data was excluded from the analyses.
Replication	All experiments were reproduced at least twice to support the conclusions stated in the manuscript.
Randomization	This work does not involve participant groups so randomization was not needed for the study.
Blinding	This work does not involve participant groups so blinding was not relevant for this kind of study.

Reporting for specific materials, systems and methods

We require information from authors about some types of materials, experimental systems and methods used in many studies. Here, indicate whether each material, system or method listed is relevant to your study. If you are not sure if a list item applies to your research, read the appropriate section before selecting a response.

Materials & experimental systems

n/a	Involved in the study	
☐	☒	Antibodies
☐	☒	Eukaryotic cell lines
☒	☐	Palaeontology
☒	☐	Animals and other organisms
☒	☐	Human research participants
☒	☐	Clinical data

Methods

n/a	Involved in the study	
☐	☒	ChIP-seq
☒	☐	Flow cytometry
☒	☐	MRI-based neuroimaging

Antibodies

Antibodies used	Western blot analyses were performed with: anti-Vinculin (V9131; Sigma-Aldrich), anti-Oct3/4 (sc5279; Santa Cruz Biotechnology), anti-Ezh2 (BD43 clone; homemade 3), anti-Suz12 (sc-46264; Santa Cruz Biotechnology), anti-Eed (AA19 clone; homemade 65), anti-p53 (homemade), anti-flag (F3165; Sigma-Aldrich), anti-Mtf2 (16208-1-AP; Proteintech), anti-Jarid2 (ab48137; Abcam), anti-Phf19 (11895-1-AP; Proteintech), anti-EPOP 66, anti-P300 (sc-585; Santa Cruz Biotechnology), anti-CBP (sc-583; Santa Cruz Biotechnology), anti-HA (12CA5 clone; homemade), anti-H3K27me1 (61015; Active Motif), anti-H3K27me2 (9728; Cell Signaling Technology), anti-H3K27me3 (9733; Cell Signaling Technology), anti-H3K27ac (ab4729; Abcam), anti-H3K9K14ac (C15410200; Diagenode), anti-H3K14ac (39599; Active Motif), anti-H3K18ac (E-AB-20285; Microtech), anti-H3K23ac (E-AB-20205; Microtech), anti-H4K5ac (39170; Active Motif, anti-H4K8ac (E-AB-20208; Microtech), anti-H4K12ac (39166; Active Motif), anti-H4 (ab7311; Abcam), anti-H2AK119ub (8240; Cell Signaling Technology), anti-H3 (1791; Abcam), and anti-H2A (12349; Cell Signaling Technology).
Validation	All the antibodies were already validated and used according to the manufacturer (or collaborators) instructions.

Eukaryotic cell lines

Policy information about cell lines

Cell line source(s)	ES-E14TG2a were obtained from IEO-IFOM Campus Transgenic Facility. Rosa26:Cre-ERT2 Ring1A KO Ring1B fl/fl conditional mESC and Ring1b KO mESC were a gift from Anton Wutz.
Authentication	Restriction analysis and Sanger sequencing were used to confirm CRSPR-based genome editing. KOs were confirmed by PCR and/or Western Blot analysis.
Mycoplasma contamination	ES-E14TG2a cells used for CRISPR editing tested negative for mycoplasma.
Commonly misidentified lines (See ICLAC register)	No cell lines used are listed in the database of commonly misidentified cell lines.

ChIP-seq

Data deposition

☒ Confirm that both raw and final processed data have been deposited in a public database such as GEO.

☒ Confirm that you have deposited or provided access to graph files (e.g. BED files) for the called peaks.

Data access links<br>*May remain private before publication.*	https://www.ncbi.nlm.nih.gov/geo/query/acc.cgi?acc=GSE116603
Files in database submission	NA
Genome browser session<br>(e.g. UCSC)	NA

Methodology

Replicates	All samples were run in biological replicates as indicated in the text
Sequencing depth	ChiP-seq and RNA-seq data were single-end, 50bp. ATAC-seq data was paired, 50bp.
Antibodies	ChIP assays were performed using: anti-Suz12 (3737; Cell Signaling Technology), anti-Ring1b (homemade; 63), anti-HA (12CA5 clone; homemade), anti-Jarid2 (ab48137; Abcam), anti-Mtf2 (16208-1-AP; Proteintech), anti-Phf19 67, anti-EPOP 66, anti-H3K27me1 (61015; Active Motif), anti-H3K27me2 (9728; Cell Signaling Technology, anti-H3K27me3 (9733; Cell Signaling Technology), anti-H3K27ac (ab4729; Abcam), anti-H3K36me3 (4909; Cell Signaling Technology), anti-H2AK119ub (8240; Cell Signaling Technology) and purified rabbit IgG (I5006; Sigma-Aldrich).
Peak calling parameters	Peaks were called using MACS2 v2.1.1 with parameters -g mm --nomodel -p 1e-10 –B
Data quality	Peaks were considered with p < 1e-10
Software	Reads were aligned to the mouse reference genome mm9 using bowtie v1.2.2 with default parameters without allowing for multi-mapping (-m 1); PCR duplicates were removed using PICARD (http://broadinstitute.github.io/picard/)
